# Therapeutic and Toxic Effects of Valproic Acid Metabolites

**DOI:** 10.3390/metabo13010134

**Published:** 2023-01-16

**Authors:** Natalia A. Shnayder, Violetta V. Grechkina, Aiperi K. Khasanova, Elena N. Bochanova, Evgenia A. Dontceva, Marina M. Petrova, Azat R. Asadullin, German A. Shipulin, Kuanysh S. Altynbekov, Mustafa Al-Zamil, Regina F. Nasyrova

**Affiliations:** 1 Institute of Personalized Psychiatry and Neurology, Shared Core Facilities, V.M. Bekhterev National Medical Research Centre for Psychiatry and Neurology, 192019 Saint Petersburg, Russia; 2 Shared Core Facilities “Molecular and Cell Technologies”, V.F. Voino-Yasenetsky Krasnoyarsk State Medical University, 660022 Krasnoyarsk, Russia; 3 Department of Psychiatry, Russian Medical Academy for Continual Professional Education, 125993 Moscow, Russia; 4 Department of Psychiatry and Addiction, Bashkir State Medical University, 45000 Ufa, Russia; 5 Centre for Strategic Planning and Management of Biomedical Health Risks, 119121 Moscow, Russia; 6 Republican Scientific and Practical Center of Mental Health, Almaty 050022, Kazakhstan; 7 Department of Psychiatry and Narcology, S.D. Asfendiarov Kazakh National Medical University, Almaty 050022, Kazakhstan; 8 Department of Physiotherapy, Faculty of Continuing Medical Education, Peoples’ Friendship University of Russia, 11798 Moscow, Russia

**Keywords:** valproic acid, blood metabolite, urinal metabolite, oxidation, glucuronidation, acetylation, pharmacometabolomics, pharmacogenomics, adverse drug reaction, risk factor, personalized approach

## Abstract

Valproic acid (VPA) and its salts are psychotropic drugs that are widely used in neurological diseases (epilepsy, neuropathic pain, migraine, etc.) and psychiatric disorders (schizophrenia, bipolar affective disorder, addiction diseases, etc.). In addition, the indications for the appointment of valproate have been expanding in recent years in connection with the study of new mechanisms of action of therapeutic and toxic metabolites of VPA in the human body. Thus, VPA is considered a component of disease-modifying therapy for multiple tumors, neurodegenerative diseases (Huntington’s disease, Parkinson’s disease, Duchenne progressive dystrophy, etc.), and human immunodeficiency syndrome. The metabolism of VPA is complex and continues to be studied. Known pathways of VPA metabolism include: β-oxidation in the tricarboxylic acid cycle (acetylation); oxidation with the participation of cytochrome P-450 isoenzymes (P-oxidation); and glucuronidation. The complex metabolism of VPA explains the diversity of its active and inactive metabolites, which have therapeutic, neutral, or toxic effects. It is known that some active metabolites of VPA may have a stronger clinical effect than VPA itself. These reasons explain the relevance of this narrative review, which summarizes the results of studies of blood (serum, plasma) and urinary metabolites of VPA from the standpoint of the pharmacogenomics and pharmacometabolomics. In addition, a new personalized approach to assessing the cumulative risk of developing VPA-induced adverse reactions is presented and ways for their correction are proposed depending on the patient’s pharmacogenetic profile and the level of therapeutic and toxic VPA metabolites in the human body fluids (blood, urine).

## 1. Introduction

**Figure 1:**
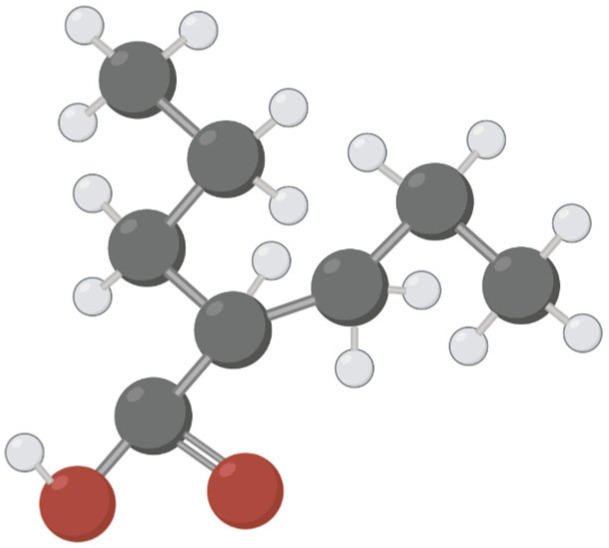
3D scheme of valproic acid molecule.

Valproic acid (CH3CH2CH2)2CHCOOH 2-propylvaleric acid, VPA) is a fatty acid derivative originally synthesized in 1881 by Beverly S. Burton. For almost a century, it has been used as a popular organic solvent in industry and pharmaceuticals [1] (Figure 1).

In 1963, George Karraz made an unexpected discovery while researching the anticonvulsant effects of hellin, finding that all samples dissolved in VPA had the same degree of antiepileptic activity. VPA (Depakene) was first approved by the United States Food and Drug Administration in 1978 [2,3]. In subsequent years, valproates began to be used for many neurological diseases and mental disorders, including: epilepsy [2]; bipolar affective disorder [4,5]; migraine [6]; neuropathic pain [7]; schizophrenia [8]; addiction to alcohol consumption [9], as well as to reduce addictive behavior and detoxification after taking narcotic substances (for example, methamphetamine) [10,11]; compulsive sexual behavior [12]; human immunodeficiency virus infection [13,14]; and progressive Duchenne muscular dystrophy [15]. Moreover, VPA has additional indications as a component of disease-modifying therapy for massive tumors due to its oncostatic effect [16].

As a rule, for most of the above-mentioned disorders, the use of VPA is chronic. This requires maintaining an optimal balance between its effectiveness and safety. This balance depends on the genetically determined features of VPA metabolism and transport in the human body [17], the dose, and duration of VPA intake. From the standpoint of metabolomics, VPA metabolites are of great interest, since they can have both the expected therapeutic effect and predicted or unexpected toxic effects [18]. Thus, toxic metabolites of VPA can lead to an increase in the severity of neurological diseases and mental disorders [19], congenital malformations in the fetus [20], dysfunction of the neuroendocrine system [21], and impaired hematopoiesis [22]. The hepatotoxic effect of VPA metabolites has been well studied [23,24].

Some of the toxic metabolites of VPA lead to disturbances in replication and deoxyribonucleic acid (DNA) synthesis [25]. However, these same metabolites can be useful in the treatment of multiple tumors [26,27,28].

The process of formation of toxic metabolic products is called “toxification”, and biotransformation products with high toxicity are referred to as toxic metabolites [29]. In some cases, the toxic VPA metabolite is an unstable compound that undergoes further transformations in the human body. In this case, it is also called an intermediate or reactive (also known as active) metabolite. It is reactive VPA metabolites that are the substances that cause damage to biosystems at the molecular level [30].

A common property of almost all reactive metabolites is their electrically deficient state, i.e., high electrophilicity [31]. Reactive toxic metabolites of VPA can interact with electron rich (nucleophilic) molecules, damaging them [32]. These nucleophilic molecules include cell macromolecules, the structure of which includes large number of oxygen, nitrogen, and sulfur atoms. These are, first of all, proteins, DNA, and ribonucleic acid (RNA) [33,34]. Reactive VPA metabolites either attach to nucleophilic molecules, forming covalent bonds with them, or cause their oxidation. In both cases, the structure of macromolecules is disturbed, and therefore their functions are also impaired [35]. Bioactivation is not always accompanied by damage to the bio-substrate, since the processes of detoxification and reparation proceed simultaneously in the human body [36]. The intensity of these two processes may be sufficient to compensate for the damage associated with the formation of reactive toxic metabolites of VPA. However, when using high doses of VPA and/or chronic pharmacotherapy, protective mechanisms may fail, leading to the development of serious VPA-induced adverse drug reactions (ADRs) [37].

The main organ of VPA metabolism in the human body is the liver, mainly due to the diversity and high activity of biotransformation enzymes there [38]. Despite the dominant role of the liver in VPA metabolism, other organs also take part in this process. The kidneys and lungs contain enzymes of I and II phases of VPA metabolism [39,40]. In addition, some of these enzymes are also expressed in the brain [41]. The role of the kidneys is particularly great because this organ has a specific system for the capture and catabolism of VPA conjugation products formed in the liver [42]. The activity of other organs, such as the brain, blood, intestine, spleen, muscle tissue, and placenta, is much lower [43,44,45,46,47]. However, genetically determined expression level and functional activity of enzymes catalyzing VPA biotransformation processes in these human organs and tissues will play a key role in the development of VPA-induced ADRs in these organs and tissues with a systemic and local elevation of reactive toxic VPA metabolites. In the process of extrahepatic metabolism of VPA, compounds (molecules) can be formed, both similar to products of hepatic origin, and different from them. In some cases, extrahepatic metabolism may activate VPA metabolites formed in the liver.

The metabolism of VPA is complicated and continues to be studied. Known pathways of VPA metabolism include: β-oxidation in the tricarboxylic acid cycle (acetylation); oxidation with the participation of cytochrome P-450 isoenzymes (P-oxidation); and glucuronidation [19,48,49]. The complex metabolism of VPA explains the diversity of its active and inactive metabolites, which have therapeutic, neutral, or toxic effects. It is known that some active metabolites of VPA may have a stronger clinical effect than VPA itself.

These reasons explain the relevance of this narrative review, which summarizes the results of studies of the blood (serum, plasma) and urinary metabolites of VPA from the standpoint of pharmacogenetics and pharmacometabolomics. We have searched full-text publications in PubMed, Web of Science, Springer, Google Scholar, and e-Library databases. Keywords were as follows: valproic acid; blood metabolite; urinal metabolite; oxidation; glucuronidation; acetylation; pharmacometabolomics; pharmacogenomics; adverse drug reaction; risk factor; and personalized approach. In addition, earlier publications of historical interest are included in the narrative review.

## 2. Mechanism of Valproic Acid Action

**Figure 2:**
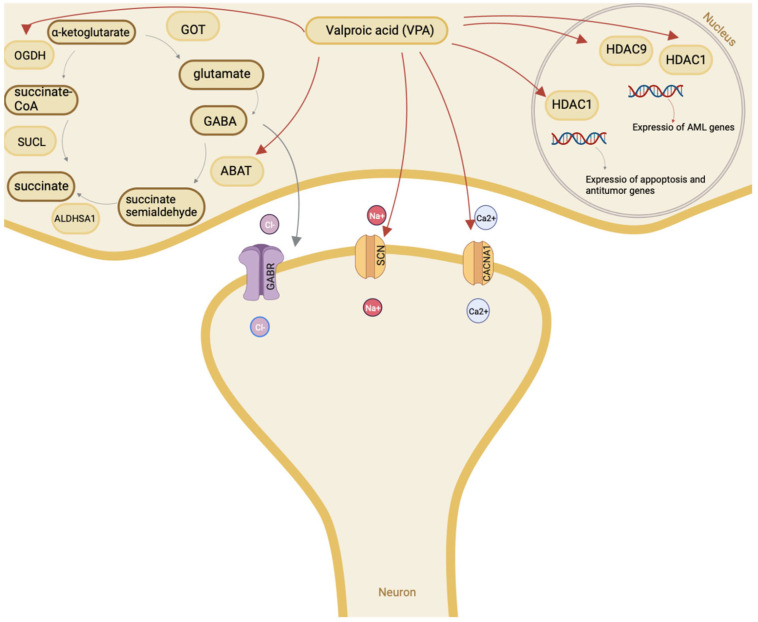
Mechanism of action of valproic acid. Note: GOT—glutamate-oxaloacetate transaminase; GABA—gamma-aminobutyric acid; ABAT—4-aminobutyrate aminotransferase; ALDHA1—aldehyde dehydrogenase; SUCL—succinate-CoA ligase; OGDH—oxoglutarate dehydrogenase; HDAC—histone deacetylase.

The exact mechanisms of action of VPA (Figure 2) and its metabolites in neurological disease and psychiatric disorders are unknown, but there are several ways to explain this. VPA and its active therapeutic metabolites are known to inhibit succinic semialdehyde dehydrogenase [49]. This inhibition leads to an increase in succinic semialdehyde, which acts as a gamma-aminobutyric acid (GABA) transaminase inhibitor, decreasing GABA metabolism and increasing GABAergic neurotransmission. Because GABA is an inhibitory neurotransmitter, this increase results in increased inhibitory activity in the central nervous system. A possible secondary factor in the inhibition of neurons in the cerebral cortex is the direct suppression of the activity of voltage-dependent sodium channels and indirect suppression of the activity of cortical neurons through the effect on GABAergic neurotransmission [50,51]. VPA and its active (therapeutic) metabolites can affect the extracellular signal-related kinase (ERK) pathway [52]. These effects are dependent on mitogen-activated protein kinase (MEK) and result in ERK1/2 phosphorylation. This MEK-induced activation increases the expression of several downstream targets, including ELK-1, followed by an increase in c-fos, a growth cone-associated protein-43 that promotes neuronal plasticity; B-cell lymphoma/leukemia-2, which is an anti-apoptotic protein; and brain-derived neurotrophic factor, which is also involved in neuronal plasticity and growth [2]. The increase in neurogenesis and neurite growth under the influence of VPA and its active metabolites is explained by the effects of this pathway. An additional effect of the VPA-induced increase in brain-derived neurotrophic factor expression is an increase in the number of GABA receptors, which contributes to an increase in inhibitory GABAergic activity [53].

VPA has a non-competitive indirect inhibitory effect on myo-inositol-1-phosphate synthetase. This leads to a decrease in de novo synthesis of inositol monophosphatase and subsequent depletion of inositol [54]. VPA and its active metabolites influence also leads to the downregulation of protein kinase C (PKC)–α and -ε proteins, which are potentially associated with bipolar disorder, since PKC is not regulated in the frontal cortex in patients with bipolar disorder [55]. Inhibition of the PKC pathway may also contribute to the prevention of migraine attacks [56]. The level of the substrate PKC is also reduced by VPA and may contribute to changes in synapse remodeling by affecting the cytoskeleton [57].

VPA and its metabolites are also known to affect fatty acid metabolism. It is believed that less incorporation of fatty acid substrates into sterols and glycerol-lipids affects the fluidity of the neuronal cell membrane and leads to an increase in the action potential threshold, which may contribute to the antiepileptic effect of valproates [58]. It has been shown that VPA is a non-competitive direct inhibitor of microsomal long-chain fatty acyl-CoA synthetase in the brain. Inhibition of this enzyme reduces the availability of arachidonyl-CoA, a substrate for the production of inflammatory prostaglandins. It is thought that this may be one of the mechanisms behind VPA’s effectiveness in migraine prevention, as migraines are usually treated with non-steroidal anti-inflammatory drugs, which also suppress prostaglandin production [59].

Finally, VPA and its active metabolites act as direct inhibitors of histone deacetylase (HDAC) [51]. Hyperacetylation of lysine residues on histones promotes the unwinding of DNA and allows for an increase in gene transcription. Such genomic effects have a wide spectrum: the activity of the 461 gene is increased or suppressed under the influence of VPA and its active metabolites (both therapeutic and toxic). The relationship of these genomic effects to the therapeutic value of VPA is not fully understood; however, hyperacetylation of H3 and H4 correlates with a decrease in the severity of bipolar affective disorder [60]. Histone hyperacetylation in the *BDNF* (Brain-Derived Neurotrophic Factor) gene, which increases brain-derived neurotrophic factor expression, occurs after an epileptic seizure and is thought to be a neuroprotective mechanism that VPA may enhance or prolong. Hyperacetylation of H3 is associated with decreased activity of glyceraldehyde 3-phosphate dehydrogenase, a pro-apoptotic enzyme, further contributing to the neuroprotective effects of VPA [61].

## 3. Pharmacometabolomics and Pharmacogenomics of Valproic Acid

Numerous studies have shown that human cytochrome P450 (CYP) isoenzymes play a crucial role in VPA metabolism [62,63]. A key CYP-mediated branch of the VPA pathway produces the metabolites 4-hydroxy(OH)-VPA and 5-OH-VPA via hepatic cytochrome P450 isoenzymes CYP2C9, CYP2B6, and CYP2A6. The use of a selective inhibitor of the CYP2C9 isoenzyme, sulfafenazole, can dramatically reduce the formation of these three metabolites; however, sulfafenazole is most likely a catalyst for the formation of 4-ene-VPA rather than 4-OH-VPA or 5-OH-VPA [5]. Isoenzymes CYP2A6 and CYP2B6 together provide the formation of 20–25% of 4-ene-VPA, 4-OH-VPA, and 5-OH-VPA. In addition, the CYP2A6 isoenzyme mediates the oxidation of VPA to 3-OH-VPA, and the use of coumarin, a potent inhibitor of CYP2A6, significantly reduces the formation of 3-OH-VPA in human liver microsomes [5].

Analysis of VPA metabolism (1 nM) in vitro showed that the common (wild) single nucleotide variant (SNV) CYP2C9*1 (wild type) of the *CYP2C9* gene, encoding a fully functional CYP2C9 isoenzyme, is associated with an active process of 4-hydroxylation and 5-hydroxylation of VPA by 75–80%. In contrast, the CYP2A6 isoenzyme contributes about 50% to the VPA 3-hydroxylation process. The role of CYP2A6 and CYP2B6 isoenzymes in the oxidative metabolism of VPA varies depending on the catalytic ability of these isoenzymes in human hepatic microsomes [64].

The carriage of low-functioning (or non-functional) and high-functioning SNVs in the *CYP2A6, CYP2B6*, and *CYP2C9* genes may partly explain the interindividual variability in VPA metabolism in the human body. Thus, patients with heterozygous or homozygous CYP2A6*4/*4 genotypes (*CYP2A6* gene deletion), known as poor metabolizers (PM), had higher plasma concentrations of VPA than extensive metabolizers (EM), homozygous carriers of the fully functional allele. Other SNVs may have a different effect on plasma concentrations of VPA and its metabolites, for example: carriers of the highly functional CYP2B6*4 allele (rs2279343; 785A>G), known as ultra-rapid metabolizers (URM), had higher activity of the CYP2B6 isoenzyme compared to carriers of the extensive variant (wild type), although protein expression was slightly reduced in patients with the URM phenotype. On the contrary, patients with the PM phenotype, who are carriers of non-functional SNVs of the *CYP2B6* gene (rs3745274, 516G>T; rs2279343, 785A>G), had a higher concentration of VPA in the blood plasma compared to patients with the EM phenotype [65].

Patients, heterozygous and homozygous carriers of the CYP2C9*3 (rs1057910; 1075A>C) and CYP2C9*2 (rs1799853; 430C>T) alleles, had an increased concentration of toxic VPA metabolites in plasma compared to homozygous carriers of the CYP2C9*1 allele [66]. It has been shown that elderly female patients require a 30–50% lower dose of VPA to achieve therapeutic concentrations of the drug compared to young men [67]. Studying the CYP2C9 profile in pediatric patients before starting treatment allowed us to optimize the dose of VPA and minimize exposure to suboptimal or toxic concentrations, thereby reducing the incidence of ADRs such as hyperammonemia. Moreover, carriers of CYP2C9*3 allelic variant, CYP2A6*1/*4 and CYP2A6*4/*4 genotypes, had elevated hepatotoxic levels of 4-en-VPA and/or 2,4-diene-VPA compared to homozygous carriers of the wild-type allele CYP2A6*1/*1 [68]. Low-functional SNVs in the *CYP2C9* and *CYP2A6* genes are risk factors for an increase in the plasma level of toxic VPA metabolites and an increase in the risk of hepatotoxicity up to 7.50 and 5.13 times, respectively. Therefore, the study of low-functioning SNVs in the *CYP2C9* and *CYP2A6* genes before treatment can predict or prevent serious VPA-induced ADRs [5].

The individual variability of alleles in the *CYP2A6*, *CYP2B6*, and *CYP2C9* genes can explain the significant variability in the pharmacokinetics of VPA and the formation of its therapeutic and toxic metabolites in different people. For example, non-functional SNVs of CYP2C9*2 and CYP2C9*3 are associated with decreased VPA metabolism, so patients who are homozygous for CYP2C9*2 or CYP2C9*3 or who are heterozygous (CYP2C9*2/*3) have a PM phenotype and show decreased P-oxidation of VPA in liver microsomes [69]. Patients with homozygous CYP2C9*2/*2 and CYP2C9*3/*3 genotype had reduced oxidative biotransformation of VPA in liver microsomes compared to subjects with compound heterozygous CYP2C9*2/*3 genotype and catalyzed the formation of toxic metabolites 4-ene-VPA, 4-OH-VPA, and 5-OH-VPA [37]. Therefore, pharmacogenetic profiling of the *CYP2C9* gene in patients with neurological diseases and psychiatric disorders may help optimize VPA dosage, and prevent ADRs and iatrogenic worsening of the disease.

In addition, CYP2C19*2 (rs4244285; 681G>A) and CYP2C19*3 (rs4986893; 636G>A) are associated with changes in the volume of distribution and plasma concentrations of VPA and its metabolites in patients with epilepsy. Thus, carriers of a highly functional CYP2C19*2 variant require higher doses of VPA to achieve target therapeutic plasma concentrations (450 μg/mL), indicating that the CYP2C19 isoenzyme is also involved in the metabolic pathway of VPA. Carriers of the CYP2C9*13 variant (rs72558187) did not require dose adjustment, as there was no correlation between the CYP2C9*13 variant and plasma VPA concentration [5]. The mean concentration/dose ratios (CDRs) of VPA were significantly higher in patients with the heterozygous CYP2C19*1/*2 genotype or compound heterozygous CYP2C19*2/*3 genotype than in patients with the homozygous CYP2C19*1/*1 genotype. The dose of VPA needs to be reduced for patients with intermediate metabolizer (IM) and PM phenotypes to achieve average blood levels of VPA in patients with the EM phenotype (CYP2C19*1/*1 genotype) [5].

A study in freshly extracted rat hepatocytes showed that VPA-induced oxidative stress and mitochondrial dysfunction preceded VPA hepatotoxicity. In addition, toxic metabolites of VPA induced hepatotoxicity by causing leakage of lysosomal membranes, as well as the formation of reactive oxygen species (ROS) as a result of metabolic activation of the CYP2E1 isoenzyme. It has been shown that the CYP2E1 isoenzyme is effective for the production of ROS and is one of the most powerful inducers of oxidative stress in cells. VPA-induced ROS formation was protected by CYP2E1 inhibitors (1-phenylimidazole and 4-methylpyrazole). In this regard, the role of the SNVs of the *CYP2E1* gene encoding this isoenzyme needs to be studied as a potentially important genetic biomarker of metabolic disorders induced by chronic valproate therapy [47].

Although CYPs play a minor role in the VPA metabolic pathway, they are important in assessing the risk of elevated plasma levels of toxic VPA metabolites, especially in patients with impaired uridine glucuronosyltransferase system (UGTs) isoenzyme activity. Due to conflicting results on the effect of CYP isoenzymes SNVs on the pharmacokinetics of VPA, larger cohorts are needed to test these results and explore new candidate genes [17].

VPA metabolism is also affected by the SNVs in the gene encoding medium chain acyl-CoA synthetase, family member 2A (*ACSM2A*). Carriers of the low-functional and non-functional SNVs of the *ACSM2A* gene have higher levels of alanine aminotransferase and aspartate aminotransferase compared to carriers of the full-functional allele (wild-type) [70], which is associated with toxic damage to hepatocytes and can lead to an increase in plasma toxic metabolites of VPA.

However, glucuronide conjugation is the predominant pathway for the metabolism and excretion of VPA [48]. Approximately 20–70% of VPA is excreted in the urine as glucuronide conjugates. Research on VPA glucuronide conjugation has focused on several genes of the *UGT1A* and *UGT2B* families, including the *UGT1A3, UGT1A4, UGT1A8, UGT1A9, UGT1A10,* and *UGT2B15* genes, as well as the best-studied *UGT1A6* and *UGT2B7* genes. It has been shown that low-functional and non-functional SNVs in the *UGT* family genes can affect the dosage and concentration of VPA in the blood plasma and can be directly related to an increase in the blood level of its toxic metabolites and the development of ADRs. A study of recombinant enzymes and human liver microsomes about the effect of three SNVs (19T>G, 541A>G, and 552A>C) in the human *UGT1A6* gene demonstrated that, compared to full-functioning SNVs (UGT1A6*1), low-functional SNVs UGT1A6*2 were associated with a 2-fold increase in VPA glucuronidation activity [71].

A study of VPA monotherapy and stable control of epileptic seizures in patients with epilepsy showed that carriers of SNVs UGT1A6*3 (rs6759892; 19T>G), UGT1A6*5 (rs2070959; 541A>G) and UGT1A6*9 (rs1105879; 552A>C) had a reduction in blood levels of VPA therapeutic metabolites. These patients required higher doses of VPA and had a lower concentration-to-dose ratio. Carriers of highly functional alleles of the *UGT1A6* gene (19T>G, 552A>C, and 541A>G) demonstrated increased activity of the UGT1A6 enzyme compared to homozygous carriers of the wild-type allele, and carriers of the 552A>C SNV in the *UGT1A6* gene had a longer half-life of toxic metabolites of VPA and a lower clearance rate, which often led to VPA-induced ADRs such as ataxia, liver damage, metabolic syndrome, tremors, hallucinations, pancreatitis, and weight gain. However, the effect of these SNVs on VPA metabolism and the development of ADRs is difficult to determine, as alterations in VPA metabolism have been described either when VPA is given alone or in combination with carbamazepine. For example, in carriers of UGT1A3*5 treated with VPA as monotherapy, a decrease in the concentration of active therapeutic metabolites of VPA in blood plasma has been described. Therefore, homozygous and heterozygous carriers of the UGT1A3*5 allele required a higher dose of VPA to reach the therapeutic range in plasma (as measured by therapeutic drug monitoring) in the range of 50–100 μg/mL [72].

The role of the *UGT2B7* gene in VPA metabolism has also been extensively studied. Several SNVs in this gene play an important role in the clearance of toxic VPA metabolites. However, studies on the association of a few SNVs, including UGT2B7*2 (802C > T; rs7439366), UGT2B7*3 (211G > T; rs12233719) or UGT2B7*4 (1192G > A; rs145725367), with plasma levels of therapeutic and toxic VPA metabolites are controversial. However, carriers of the homozygous and heterozygous genotypes (TT and CT, respectively) UGT2B7*2 had lower plasma concentrations of VPA compared to carriers of the homozygous CC genotype, suggesting that carriers of the TT and CT genotypes may require higher doses of VPA [5]. Furthermore, patient age has been shown to be positively correlated with adjusted serum VPA concentrations in patients with pediatric epilepsy. Thus, carriers of UGT1A3*5 require a higher dose of VPA to achieve the reference (therapeutic) corridor in the plasma in the range of 50–100 μg/mL [73]. In addition, SNVs in the *UGT1A3* gene affect plasma levels of VPA therapeutic metabolites [74].

Drug transporter proteins play a critical role in the pharmacokinetics of VPA. Overexpression of efflux drug transporter proteins can also be regulated by the pregnane X receptor (PXR) [75]. Among them, the most studied VPA transporter is P-glycoprotein (P-gp) or multidrug resistance protein. P-gp is a volatile efflux pump that eliminates the action of several active metabolites’ VPA and is a product of the ATP-binding cassette subfamily b member 1 (*ABCB1*) gene, also known as the multidrug resistance gene 1 (*MDR1*) [76]. The *ABCB1* gene is one of the most important genetic biomarkers affecting drug transport across the brain–blood barrier. Like *ABCB1*, *ABCC2* gene expression is higher in brain endothelial cells of patients with VPA resistance [77]. It is known that SNVs in the *ABCB1* gene can directly affect the uptake of VPA by the brain and also the excretion (efflux) of VPA from the brain into the blood. Homozygous TT genotypes of G2677 and C3435 polymorphisms, as well as TT, CTT, and TTT haplotypes of the *ABCB1* gene, are significantly associated with the development of therapeutic resistance to VPA. At the same time, these more often had the *ABCB1* 3435 CC and CT genotypes. Such patients had reduced plasma levels of VPA therapeutic metabolites compared to the homozygous TT genotype [78,79]. In patients with temporal lobe epilepsy, an association was found between therapeutic resistance to VPA and the CGC haplotype, as well as with the CC or CT genotypes of two SNVs (rs1045642 and rs1128503) of the *ABCB1* gene [80]. The results of a meta-analysis [81] demonstrated that the SNV G2677T>A of the *ABCB1* gene is a biomarker for an increased risk of developing therapeutic resistance to valproate.

Five key enzymes are involved in the urea cycle, including carbamoyl phosphate synthetase 1 (SPS1), ornithine transcarbamoylase, argininosuccinate synthase, argininosuccinate lyase, and arginase 1. Another N-acetylglutamate synthase (NAGS) expressed in mitochondria is also important for the functioning of the urea cycle as it provides the necessary mutant N-acetylglutamic acid activator. A study in rat liver mitochondria showed that the valproil-CoA metabolite inhibits NAGS activity, leading to ammonia accumulation. Thus, CPS1 is the first limiting enzyme in the urea cycle, which accelerates the conversion of ammonium to carbamoyl phosphate in the liver. Dysregulation of CPS1 function may result from genetic changes or epigenetic regulation in hepatocellular carcinoma. Therefore, it can be assumed that the absence of CPS1 or a decrease in its activity may be largely associated with VPA-induced hyperammonemia. The 4217C > A polymorphism of the *CPS1* gene leads to the amino acid conversion of threonine to asparagine, which correlates with the low functional activity of the CPS1 enzyme [82]. This SNV has been shown to be a risk factor for VPA-induced hyperammonemia [24,83] and the development of hyperammoniemic encephalopathy.

In addition, significant impairments in mitochondrial oxidation of toxic VPA metabolites are observed in patients with SNVs of the DNA polymerase gamma, catalytic subunit (*POLG)* gene encoding polymerase γ. The POLG enzyme is defined as a mitochondrial DNA polymerase that is associated with various metabolic disorders and monogenic hereditary diseases such as Alpers–Huttenlocher syndrome, which is associated with an increased risk of fatal hepatotoxicity of VPA metabolites [84,85]. Approximately one third of patients with Alpers–Huttenlocher syndrome develop liver failure within 3 months of starting VPA medications. Low-functional SNVs in the *POLG* gene occur in up to 0.5% of the population [86]. In addition, chronic administration of VPA drugs leads to significant overexpression of the *POLG* gene and an increase in mitochondrial biogenesis by altering the expression of several mitochondrial genes. Heterozygous mutations p.Q1236H and p.E1143G in the *POLG1* gene have been associated with VPA-induced liver failure. Therefore, pharmacogenetic testing (PGx) of SNVs and mutations in the *POLG* gene is recommended in all patients with suspected mitochondrial disease prior to initiation of VPA treatment. In addition, PGx is useful in all patients with VPA-induced ADRs to rule out their association with the carriage of low-functioning (or non-functional) SNVs in the *POLG1* gene. This approach will minimize the risk of liver failure in patients receiving valproate [85,87].

A significant increase in the level of γ-glutamyltransferase (GGT) was shown in patients with heterozygous (GSTM1/GSTM1^-^) and homozygous (GSTM1^-^/GSTT1^-^) genotypes in patients treated with VPA. The increase in GGT enzyme activity in VPA-treated patients may be due to hepatic glutathione (GSH) elimination and has also been presented as an early biomarker of oxidative stress. However, the clinical relationship between the level of GGT and the SNVs in the *GST* gene and the formation of toxic metabolites of VPA remains unknown. At the same time, an association was found between the homozygous Val/Val genotype in the *SOD2* (Superoxide Dismutase 2) gene with an increase in the level of alanine aminotransferase (OR = 3,5; *p*-value = 0.056) caused by hepatotoxic metabolites of VPA [88].

In addition to hepatotoxicity, VPA metabolites also cause teratogenicity by downregulating *IGF2R* (Insulin-like growth factor 2 receptor), *RGS4* (Regulator of G protein signaling 4), *COL6A3* (Collagen, type VI, alpha 3), *EDNRB* (Endothelin B receptor), and *KLF6* (Krueppel-like factor 6) genes, as demonstrated in VPA-induced neural tube defects in a chick embryo prevalence model [89]. The *116C/G* variant (rs226957) in the promoter region of the *XBP1* (X-Box Binding Protein 1) gene is associated with increased response to active VPA metabolites; in patients carrying the minor G allele, the expected therapeutic response to VPA increased [90].

It has also been shown that SNV rs1137101 of the *LEPR* (Leptin Receptor) gene, rs1800497 of the *ANKK1* (Ankyrin Repeat and Kinase Domain Containing 1) gene, and rs10789038 of the *PRKAA2* (Protein Kinase AMP-Activated Catalytic Subunit Alpha 2) gene are associated with VPA-induced weight gain. The administration of valproate to obese patients results in an increase in serum leptin levels compared to non-obese patients [91], but there is no answer yet as to which VPA metabolites induce this ADR. However, it is known that VPA and its active toxic metabolites can reduce the expression of the up-regulated gene 4/up-regulator of cell proliferation *URG4/URGCP (URG4/URGCP)* and *CCND1* (Cyclin D1) genes and suppress the proliferation of neuroblastoma cells is a human-derived line cell used in scientific research SHY5Y [27,92,93].

Thus, pharmacogenomics and pharmacometabolomics can be the most important sources for assessing interindividual differences in the pharmacokinetics of VPA and its metabolites. Optimization of valproate dosage based on the results of pharmacogenetic and pharmacometabolic testing plays an important role in psychopharmacotherapy, so the study of genetic biomarkers that affect the pharmacokinetics of VPA can help develop a new personalized strategy for prescribing valproates to patients with neurological diseases and mental disorders by achieving a balance of efficacy and safety [17].

## 4. Therapeutic Metabolites of Valproic Acid

**Figure 3:**
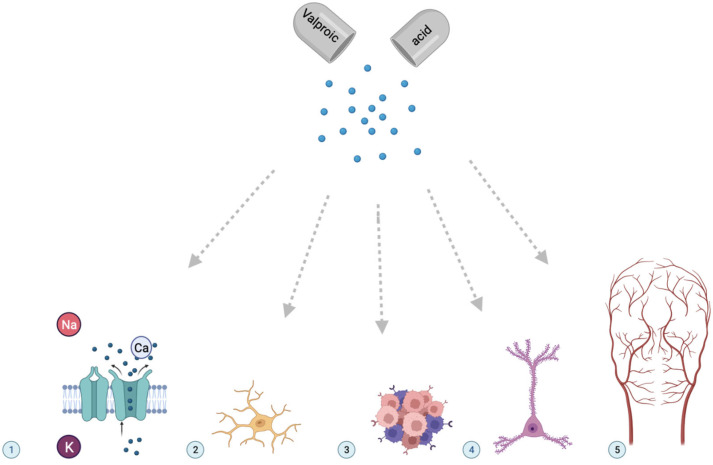
The most common clinical therapeutic effects of valproic acid metabolites. Notes: (1) blockade of voltage-dependent sodium, calcium, and potassium channels; (2) neuroprotection; (3) inhibition of signaling pathways in cancer cells; (4) modulation of neurogenesis; (5) angioprotection.

A metabolite is any substance formed during metabolism (digestion or other chemical processes in the body) [94]. In addition, the term “metabolite” refers to substances that remain (circulate in the blood, accumulate in organs and tissues, excreted in the urine) after the drug is broken down (metabolized) by the body [95]. Research into therapeutic metabolites of VPA has been ongoing for many years. Such studies have intensified over the past decade due to the rapid development of a new direction in personalized medicine—pharmacometabolomics [96]. The number of studied VPA metabolites is close to fifty [97], but their clinical role (Figure 3) in the expected therapeutic response to valproate has not yet been sufficiently studied for many of them.

The most studied therapeutic metabolites of VPA in the blood and urine are presented in Table 1 and Table 2.

### 4.1. Valproate Acid Glucuronide

Valproate acid glucuronide (VPAG) is a glucuronidation product of VPA. It is known that VPAG is the main metabolite of VPA excreted in the urine (30–50% of the dose). With the age of the patient, the level of this metabolite in the blood does not change, despite the known decrease in liver weight [97].

### 4.2. 2-N-Propyl-2-Pentenoic Acid

2-n-propyl-2-pentenoic acid is also known as 2,4-diene VPA. This VPA metabolite is formed in the body by glucuronidation. This therapeutic metabolite of VPA exhibits a high antiepileptic potential similar to VPA, but it has a low teratogenic potential [102], which is of undoubted clinical interest.

### 4.3. Valproyl-CoA

Valproyl-CoA is a therapeutic metabolite of VPA, which is formed by the metabolism of VPA as a result of β-oxidation (acetylation) in the Krebs cycle [100]. Previously, this metabolite was found in liver cells (hepatocytes), but recent work has shown that valproyl-CoA can accumulate in brain neurons as a result of normal fatty acid turnover processes. Valproil-CoA is a therapeutic metabolite and increases the antiepileptic activity of VPA by stimulating the activity of Na+, K+-ATPase at low concentrations of adenosine triphosphate in the brain [100].

### 4.4. 4-Ene-Valproic Acid

It is known that 4-ene-valproic acid (4-ene-VPA) belongs to the class of organic compounds known as methyl-branched fatty acids. These are fatty acids with an acyl chain having a methyl branch. Usually, they are saturated and contain only one or several methyl groups [101]. However, other branches than the methyl branch may also be present. In addition, 4-ene-VPA is a very hydrophobic molecule; it is practically insoluble (in water) but refers to relatively neutral VPA metabolites, although this opinion is debatable. Several studies have demonstrated that 4-ene-VPA has an antiepileptic effect and also inhibits HDACs, which is a promising direction in the treatment of cancer, autoimmune disease, and acquired immunodeficiency syndrome [97]. 4-ene-VPA circulates in the blood and is excreted in the urine, which facilitates the study of its level in biological fluids of the human body.

### 4.5. 2-Ene-Valproic Acid

2-ene-valproic acid (2-ene-VPA) is a product of P-oxidation metabolism in hepatocytes. This therapeutic metabolite of VPA belongs to a class of organic compounds known as methyl-branched fatty acids. In addition, it inhibits HDACs, which is a promising direction in the treatment of cancer, autoimmune disease, and acquired immunodeficiency syndrome [101].

## 5. Role of Therapeutic Metabolites of Valproic Acid

Thus, VPA is a derivative of fatty acids and HDACs of classes I and IIa [103], which allows VPA and its active metabolites to regulate the expression of various genes [103]. In addition, pathophysiological processes include both excitotoxicity of VPA metabolites and a decrease in gene transcription due to a decrease in the level of histone acetylation, which is accompanied by a loss of GABAergic (gamma-aminobutyric acid) neurons in the striatum as a pathological hallmark of Huntington’s disease [13,104]. VPA is known to exert neuroprotective effects through these primary targets. Excitotoxicity is a mechanism of cell death caused by hyperactivation of excitatory amino acid receptors, which increases the ion permeability of the cell membrane and leads to an overload of intracellular calcium [105]. Notably, VPA and its active metabolites also strongly induce 70 kilodalton heat shock proteins through epigenetic mechanisms, reducing infarct volume and improving functional recovery in rodents subjected to cerebral ischemia [106]. HDAC1 has been implicated in reducing the human immunodeficiency viruses’ activity in infected cells. The inhibitory effect of VPA metabolites on this protein makes it a good candidate for acquired immunodeficiency syndrome therapy [107].

Neuroprotective effects of VPA therapeutic metabolites have been reported in a wide range of acute central nervous system injury models, including post-traumatic inflammation caused by spinal cord injury [108]. The results of the studies demonstrated that VPA and its therapeutic metabolites promote the phenotypic shift of microglia from the M1 phenotype to the M2 phenotype and inhibited microglial activation, thereby reducing the level of pro-inflammatory factors caused by spinal cord injury. The increase in acetylation of signal transducer and activator of transcription 1/ nuclear factor-κB (STAT1/NF-κB pathway) during VPA treatment was probably caused by HDAC3 translocation to the nucleus and its activity. In addition, VPA and its therapeutic metabolites have been shown to downregulate HDAC3 expression and activity and increase acetylation of STAT1 as well as NF-κB p65 after spinal cord injury. The acetylation status of NF-kB p65 and the complex with nuclear factor kappa-light-chain-enhancer of activated B cells NF-κB (NF-κB) p65 and STAT1 suppressed the transcriptional activity of NF-kB p65 and attenuated the central inflammatory response mediated by microglia and led to the neuroprotective effect of VPA therapeutic metabolites [109].

As mentioned earlier, VPA has been described as an HDAC, which makes it possible to consider not only the well-known therapeutic metabolites of VPA, but also the toxic metabolites of VPA, as promising potential therapeutic agents for the treatment of various forms of cancer and leukemia [110]. Chromatin is formed from DNA packaged in nucleosome structures of 146 base pairs of DNA wound around a histone octamer (two copies of each histone: H2A, H2B, H3, and H4) held in place by histone H1. In terms of transcription, the condensed form of chromatin (heterochromatin) is inactive. In contrast, the decondensed form (euchromatin) corresponds to the active form. The transition between euchromatin and heterochromatin depends on two families of proteins: histone acetyltransferases and HDACs. It has been established that histone acetylation leads to relaxation of the nucleosome structure, releasing DNA and allowing activation of transcription. Inhibition of HDAC promotes the formation of decondensed chromatin, thereby promoting gene expression [13]. The toxic metabolites of VPA are among the most promising molecules because, unlike other drugs that target the expression of a molecule or family of molecules, they target chromatin through associated proteins: HDAC; deoxyribonucleic acid methyltransferase; heterochromatin protein 1; and smooth muscle cells. Thus, toxic VPA metabolites affect the expression of many proteins and therefore can be considered as therapeutic and applicable to a wide range of neurodegenerative diseases and autoimmune diseases, as well as multiple tumors and leukemia, where these VPA metabolites repress many antioncogenes during carcinogenesis [13].

Parkinson’s disease is caused by the degeneration of nigrostriatal dopaminergic neurons. No direct effect of VPA on Parkinson’s disease has been shown, but in vitro studies have shown a positive effect of active VPA metabolites in animal models that mimic Parkinson’s disease at various levels. VPA and its active metabolites prevent rotenone-induced apoptosis, protect neurons from the dopaminergic toxin 1-methyl-phenylpyridinium, stimulate the release of neutrophils from neuroglial cells, protect cultured dopaminergic neurons from degeneration caused by excessive microglial activation by stimulating microglial apoptosis, and protect neurons, increasing the expression of α-synuclein and preventing its monoubiquitination and nuclear translocation [13]. Experiments on an animal model of Parkinson’s disease (rodents) have shown that selective changes in α-synuclein induced by rotenone (a decrease in native protein and an increase in monoubiquitination in substantia nigra and striatum) can be reduced with long-term administration of VPA [104]. However, there is still no convincing answer to the question about which of the active metabolites of VPA are the most promising molecules for the treatment of Parkinson’s disease and other neurodegenerative diseases, including Huntington’s disease and Alzheimer’s disease.

The role of active VPA metabolites in the treatment of primary muscular dystrophy (eg., Duchenne muscular dystrophy) has been demonstrated in vitro and in vivo in *mdx/utrn^-/-^* knockout mice. It has been shown that VPA therapeutic metabolites are able to induce the Akt/mTOR/p70S6K pathway through the induction of phosphatidylinositol-3-OH kinase, which leads to a decrease in collagen content and fibrosis in skeletal muscles, a decrease in hind limb contractures, an increase in the integrity of the myocyte sarcolemma, a decrease in the number CD8-positive inflammatory cells, and an increase in the level of activated Akt in the skeletal muscles of experimental animals [13].

Additionally, therapeutic metabolites of VPA have the potential to delay the development of atrial remodeling in patients from the risk group. Therapeutic metabolites of VPA have been shown to attenuate many components of atrial remodeling that are present in transgenic mice, animal models, and humans. However, VPA therapeutic metabolites significantly reduced atrial dilatation, cardiomyocyte enlargement, atrial fibrosis, and myocyte ultrastructure disorganization. In addition, it was demonstrated that these metabolites significantly reduced the occurrence of atrial thrombi and reduced the severity of negative changes in the action potential of cardiomyocytes [111]. An increase in H4 histone acetylation in the atria of VPA-treated transgenic mice confirmed the efficacy of HDAC inhibition in vivo. Cardiomyocyte-specific genetic inactivation of HDAC2 in transgenic mice attenuated ultrastructural myocyte disorganization comparable to VPA. Finally, in transgenic mice, VPA therapeutic metabolites restrain dysregulation of proteins that are involved in multiple pathways associated with atrial fibrillation, such as oxidative phosphorylation or RhoA (Ras homolog gene family, member A) signaling, and disease functions such as cardiac fibrosis and muscle cells apoptosis [111].

## 6. Toxic Metabolites of Valproic Acid

The toxicity of VPA metabolites refers to the level of damage these compounds can cause to the human body. The toxic effects of VPA metabolites are dose-dependent and may affect the entire system, such as the central nervous system, or a specific organ, such as the liver.

**Figure 4:**
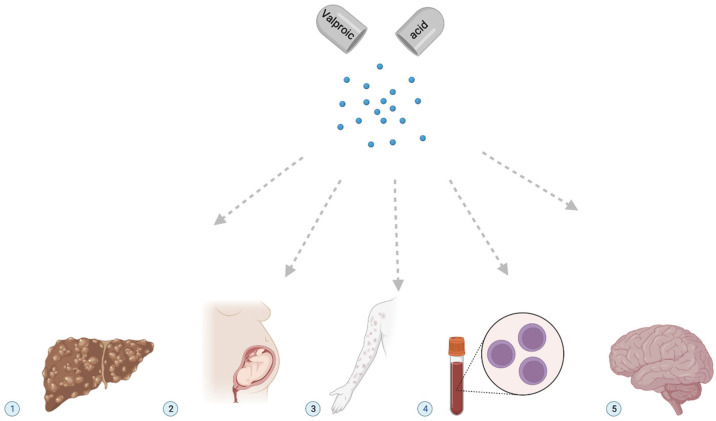
The most common clinical effects of toxic metabolites of valproic acid. Notes: (1) hepatotoxicity; (2) teratogenicity; (3) dermatitis, alopecia; (4) thrombocytopenia; (5) depression of the central nervous system.

Toxic metabolites of VPA can form in the human body as intermediates, byproducts, or end products of its metabolism. There are many examples of how hepatocytes organize metabolic processes in space and time to prevent the accumulation of these toxic VPA metabolites or reduce their level in organs and tissues in which biological processes that are most sensitive to toxic VPA metabolites occur [13] (Figure 4).

The most studied toxic metabolites in the blood (serum and plasma) and urine are presented in Table 3 and Table 4.

### 6.1. 3-Hydroxyvalproic Acid

3-Hydroxyvalproic acid (3-OH-VPA) belongs to a class of organic compounds known as hydroxy fatty acids [97]. These fatty acids do have a hydroxyl group in their chain. This VPA metabolite is formed in the body, mainly in hepatocytes, as a result of P-oxidation involving two isoenzymes: CYP3A5 and CYP2A6. This VPA metabolite has a pronounced hepatotoxic effect [97], which explains the importance of PGx (Appendix A) for identifying patients at risk.

### 6.2. 4-Hydroxyvalproic Acid

4-Hydroxyvalproic acid (4-OH-VPA) also belongs to the class of organic compounds known as hydroxy fatty acids. These are fatty acids that have a hydroxyl group in their chain. This toxic metabolite is the result of P-oxidation of VPA in hepatocytes (primarily) by four isoenzymes: CYP3A5; CYP2A6; CYP2C9; and CYP2B6. It is known that 4-OH-VPA has a pronounced hepatotoxic effect [97], which explains the importance of PGx (Appendix A) for identifying patients at risk.

### 6.3. 5-Hydroxyvalproic Acid

5-Hydroxyvalproic acid (5-OH-VPA) belongs to a class of organic compounds known as hydroxy fatty acids. These are fatty acids that have a hydroxyl group in their chain. This toxic metabolite of VPA is formed in the human body (mainly in hepatocytes) by P-oxidation involving three cytochrome P450 isoenzymes: CYP2A6; CYP2C9; and CYP2B6 [97]. 5-OH-VPA has been shown to have a hepatotoxic effect, which also explains the importance of PGx (Appendix A) in identifying patients at risk.

### 6.4. 3-Oxovalproic Acid

3-Oxovalproic acid belongs to a class of organic compounds known as short-chain keto acids and their derivatives [97]. These keto acids do have an alkyl chain containing fewer than six carbon atoms. This toxic metabolite of VPA has a suspected toxic effect [97], and studies of its toxic potential are ongoing.

### 6.5. Valproyl-CoenzymeA

Valproil-CoA is the main metabolite of VPA produced in mitochondria. In addition, valproyl-CoA affects the activity of succinate-CoA ligase, especially the ATP-specific enzyme [115]. Inhibition of activated succinate-CoA ligase can affect nucleoside-diphosphate kinase activity, causing an imbalance of nucleotides in mitochondria and, ultimately, depletion of mitochondrial DNA [115]. This may explain the hepatotoxic potential of this VPA metabolite and the development of liver failure in patients with a deficiency in the mitochondrial DNA replicase system, such as gamma polymerase [115].

### 6.6. 2-N-Propyl-4-Oxopentanoic Acid

2-N-propyl-4-oxopentanoic acid belongs to a class of organic compounds known as gamma-keto acids and their derivatives. These are organic compounds containing an aldehyde substituted by a keto group on the C4 carbon atom [114]. It has been previously shown that 4-keto-VPA causes abnormal and irregular growth of the neural tube neuroepithelium, microvesicular steatosis, and reduces ammonia excretion through inhibition of carbamoyl phosphate [114], which significantly increases the risk of VPA-induced ADRs, including teratogenesis.

### 6.7. 4-Ene-Valproic Acid

4-Ene-valproic acid (4-ene-VPA) belongs to a class of organic compounds known as methyl-branched fatty acids [97]. These are fatty acids with an acyl chain having a methyl branch. They are usually saturated and contain only one or a few methyl groups. However, branches other than the methyl branch may also be present. It is formed in the body as a result of P-oxidation of valproates in the liver with the participation of isoenzymes CYP2A6, CYP2C9, and CYP2B. As previously stated, 4-ene-VPA is a highly hydrophobic molecule that is considered a toxic metabolite of VPA in patients with epilepsy and psychiatric disorders; this metabolite has a hepatotoxic effect, causing hyperammonemia and Reye’s syndrome in rats [97,114]. At the same time, 4-ene-VPA is being actively studied as a potential therapeutic metabolite in multiple tumors, autoimmune diseases, and AIDS [114].

### 6.8. 2-Propyl-2,4-Pentadienoic Acid

2-Propyl-2,4-pentadienoic acid (also known as 2,4-diene-VPA) is metabolized in the human body by glucuronidation [97]. This toxic metabolite of VPA binds reversibly to the α-subunit of the trifunctional protein, inhibiting β-oxidation. As a result, 2,4-diene VPA reduces levels of acetyl-CoA and glutamate, which are required for the synthesis of N-acetylglucosamine 6-phosphate deacetylase. A decrease in the level of N-acetylglucosamine 6-phosphate deacetylase, a cofactor for the CPSI enzyme, leads to impaired urea production and the subsequent development of VPA-associated hyperammonemia and hyperammonemic encephalopathy, cerebral edema [68]. This VPA metabolite is formed in the body by P-oxidation with the help of CYP2A6 and CYP2C9 isoenzymes [114].

### 6.9. 2-Ene-Valproic Acid

2-Ene-valproic acid (2-ene-VPA) is a product of VPA metabolism in the liver by β-oxidation. This toxic metabolite of VPA belongs to a class of organic compounds known as methyl-branched fatty acids [97]. It is known that 2-ene-VPA is a neurotoxic metabolite of VPA [114]; therefore, an increase in the level of this metabolite in the blood and in the central nervous system can lead to VPA-induced neurological ADRs (diplopia, ataxia, seizures) and mental ADRs (cognitive disorders, behavioral disorders).

### 6.10. Valproylcarnitine

Valproyl-carnitine belongs to a class of organic compounds known as acylcarnitines [116]. These are organic compounds containing a fatty acid, the carboxylic acid of which is attached to carnitine through an ester bond. VPA reduces the concentration of L-carnitine in the body through the excretion of their acyl-carnitines, pivaloyl-carnitine, and valproyl-carnitine in the urine [113], which causes the development of such ADRs as: lipid myopathy; hypoglycemia; fatty liver disease; and hyperammonemia.

## 7. Role of Toxic Metabolites of Valproic Acid

As is known, the toxicity of VPA and its metabolites refers to how toxic or harmful these substances can be and which organ(s) or tissue(s) are most sensitive to their toxic effects (Figure 4). This occurs when there are too many toxic VPA metabolites in the blood as a result of a primary (genetically determined) disturbance of drug metabolism, which leads to the ADRs of toxic metabolites on the human body. In addition, an increase in the level of toxic metabolites of VPA in biological fluids, organs, and tissues can occur when too high a dose of VPA is administered, when there are secondary VPA metabolism violations in the liver (for example, in elderly patients or in patients with liver failure), or when there is a violation of toxic VPA metabolites excretion from the bloodstream through the kidneys as a result of their primary or secondary breakdown.

One of the reasons for the development of ADRs of toxic VPA metabolites is the induced carnitine deficiency due to the excretion of valproyl-carnitine by the urine [117] and, as a consequence, impaired mitochondrial oxidation [118]. In addition, some VPA metabolites are toxic to isolated liver mitochondria with an effect similar to VPA accumulation [119]. As a result, hepatic microvesicular steatosis develops, as well as hyperammonemia, anion gap acidosis, metabolic encephalopathy, and hepatotoxicity [24]. Toxic VPA metabolites also induce teratogenicity by downregulating the *IGF2R, RGS4, COL6A3, EDNRB*, and *KLF6* genes, which are related to neural tube defects [90] in children whose mothers took valproate during pregnancy [120].

In addition, the co-administration of other drugs can lead to an increase in the level of toxic metabolites of VPA in the blood. For example, acetylsalicylic acid increases plasma levels of VPA and its toxic metabolites by competing for plasma protein binding [121,122]. This is demonstrated in a study where the authors profiled VPA and VPA metabolites in urine before (day 1) and after (day 2) administration of antipyretic doses of acetylsalicylic acid in seven subjects with stable serum VPA levels according to therapeutic drug monitoring. Of the 13 metabolites analyzed by gas-liquid chromatography / mass spectrometry, levels of (E)-2-ene-VPA and 3-keto-VPA decreased significantly on day 2, while levels of VPA (glucuronide) and 4-ene-VPA went up significantly. VPA β-oxidation pathway activity, including (E)-2-ene-VPA, 3-OH-VPA and 3-keto-VPA, decreased from 24.5% ± 10.3% of total metabolites excreted on day 1, up to 8.3% ± 4.2% on day 2. Thus, a slowdown in VPA metabolism was shown with the additional appointment of acetylsalicylic acid by 66% of the original. At the same time, the content of VPA glucuronide increased from 50.5 ± 12.6% on day 1 to 65.5 ± 14% of the total amount of excreted metabolites on day 2, which is 30% more than the initial value. The ratio of VPA glucuronide “day 2 / day 1” was statistically significantly correlated with the ratio of the average free fraction of VPA “day 2/day 1” (r = 0.9424; *p*-value = 0.005). Inhibition of VPA β-oxidation by salicylate was sufficient to counterbalance the increased elimination of VPA in the form of its conjugates and explain why the total clearance of VPA after administration of this salicylate remains unchanged, even if the free fraction of VPA increases. Metabolic profiles indicate that salicylate probably inhibits VPA β-oxidation by reducing the formation of valproil-CoA [123]. However, such a toxic effect of the combination of acetylsalicylic acid and VPA may have a synergistic killing effect on the proliferation and apoptosis of hepatocellular cancer cells [124]. These studies open new perspectives and re-evaluate the clinical role of toxic VPA metabolites.

Also, toxic metabolites of VPA can cause drug-induced parkinsonism. This VPA-induced neurological ADR occurs equally in males and females, but is more common in the elderly (usually over 55 years of age) and is not dose-dependent [125]. According to previous studies, in all patients with VPA-induced parkinsonism, serum levels of VPA were in the therapeutic range (50–100 µg/mL), although the level of neurotoxic metabolites of VPA was not investigated. Chronic maintenance doses of VPA appear to result in increased levels of its toxic metabolites in the central nervous system, which is the main cause of this ADR. This hypothesis is supported by the results of studies demonstrating that the symptoms of VPA-induced parkinsonism usually decrease within a few weeks and completely disappear within a few months after stopping valproate. However, if extrapyramidal symptoms do not improve, then it is likely that the toxic metabolites of VPA have unlocked the hidden potential (genetic predisposition) for the development of Parkinson’s disease. Such patients may benefit from the addition of levodopa therapy [126], although the mechanism of how toxic VPA metabolites cause the development of parkinsonism remains unknown [127].

## 8. Discussion

The results of the technical and scientific advances of the 21st century demonstrate the astonishing advances that have made it possible to detect, identify, and quantify a large number of VPA metabolites in biological fluids and/or tissues of the human body. In addition, similar technical and scientific advances have occurred in DNA sequencing. These advances explain the importance of moving beyond the study of individual VPA metabolites and individual genetic polymorphisms to the study of probably hundreds of VPA metabolites and even more genomic variants in a single cell or subject. In recent decades, it has become possible to combine and integrate large datasets from various “-omics” methods to improve our understanding of the molecular basis of VPA-induced ADRs risks and/or drug response phenotypes to VPA and its therapeutic and toxic metabolites.

Undoubtedly, the new concept that metabolomic profiles (metabolotypes) can help predict the phenotypes of the expected therapeutic response to VPA and its therapeutic and toxic metabolites [128] seems to be relevant and important from a clinical point of view. Obviously, the variation in metabotype among patients taking valproate reflects variations due to both environmental factors (eg., a dose of valproate or duration of therapy) and genetic factors. As a result, pharmacometabolomics may provide the practicing neurologist, psychiatrist, and other professionals (eg., pharmacologist, oncologist, or hematologist) with a better understanding of the overall molecular variability that contributes to individual differences in therapeutic response to valproate than pharmacogenomics alone [129].

**Figure 5:**
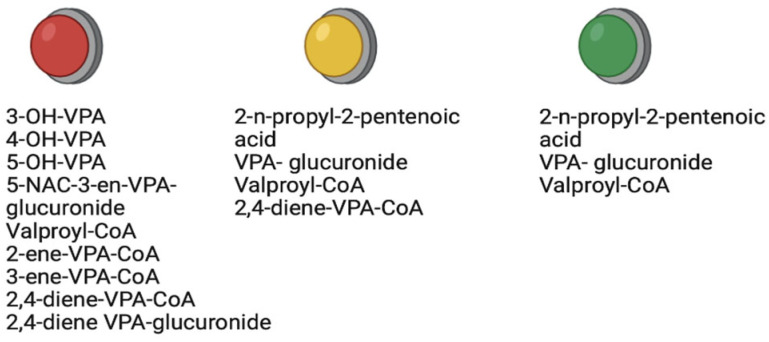
Clinical classification of valproic acid metabolites: green sign–therapeutic metabolites; yellow sign–neutral metabolites; red sign–toxic metabolites.

Thus, the pharmacogenomics and pharmacometabolomics of VPA are closely interconnected. Known genetic biomarkers of changes in VPA metabolism are summarized by us and presented in Appendix A. VPA metabolites can be divided into three groups: therapeutic, neutral, and toxic (Figure 5). However, such an assessment should be considered conditional, since the effect of VPA metabolites will depend on the goal of the therapeutic strategy for a particular disease in a particular patient (individual). This is important to remember because the same VPA metabolite can be considered both therapeutic (e.g., in massive tumors and neurodegenerative diseases) and toxic (e.g., in congenital malformations of the fetus in women taking VPA during pregnancy).

**Figure 6:**
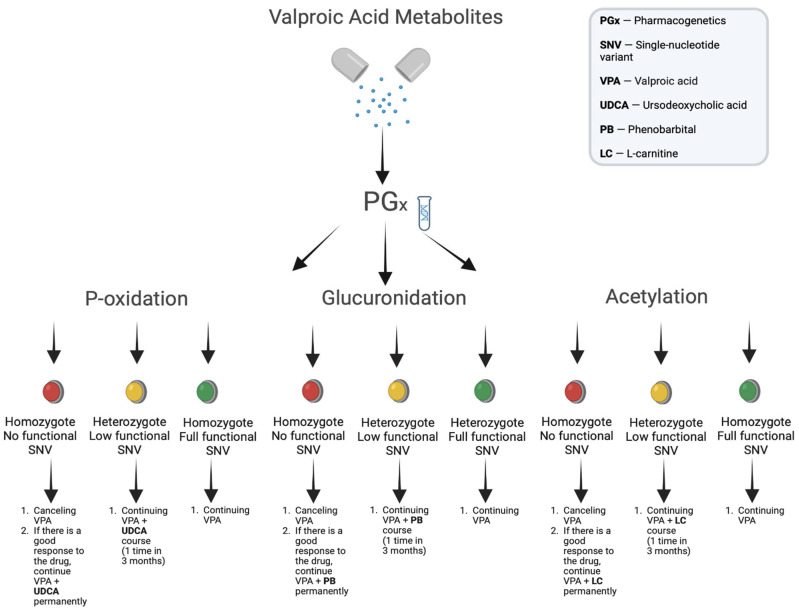
Pharmacogenomic approach to assessing the risk of development- and correction-adverse reactions induced by valproic acid metabolites.

However, despite the fact that the application of an integrated (pharmacogenomics + pharmacometabolomics) approach represents significant progress in the last decade, most researchers and clinicians are still focused on using only one -omics method in real clinical practice using VPA without evaluating the potential contribution of various -omics with potential new ideas that could provide such a combined approach [128]. For example, pharmacogenetics focusing on individual genes and their influence on the expected drug response to valproate and other drugs, as well as the assessment of individual (personalized) risk of VPA-induced ADRs (Figure 6), has turned into pharmacogenomics using genome-wide studies of clinical drug phenotypes [130].

**Figure 7:**
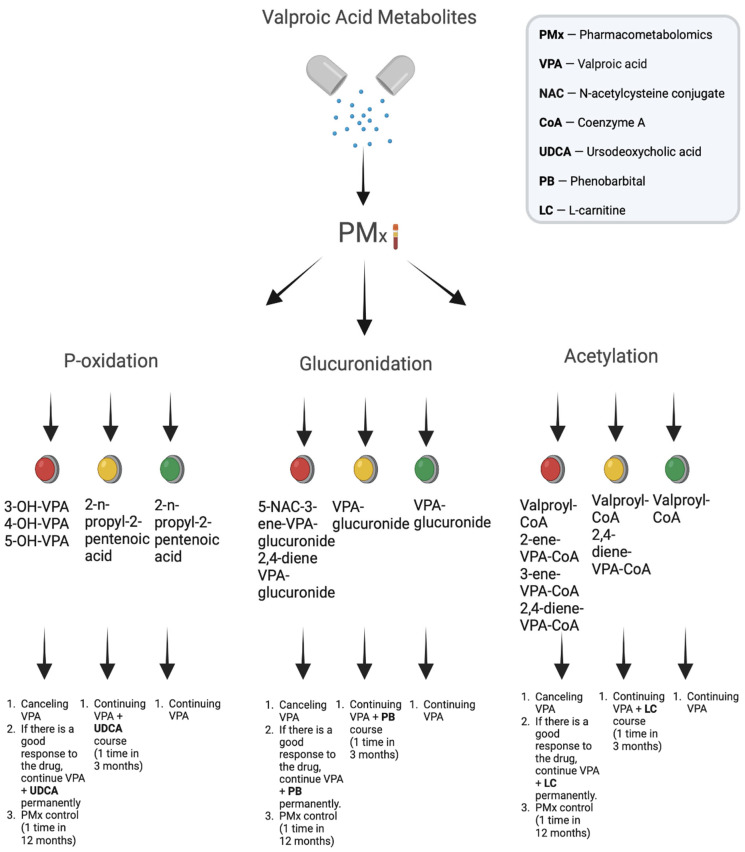
Pharmacometabolomic approach to assessing the risk of development and correction of adverse reactions induced by valproic acid metabolites.

At the same time, the addition of data from other studies, i.e., the further evolution of pharmacogenomics into “pharmacoomics”, including the pharmacogenomics and pharmacometabolomics of VPA (Figure 7), is now possible and may be required in real clinical practice, especially for studies of drug therapy with VPA drugs for complex diseases with many different underlying issues at the basis of pathophysiological mechanisms, which include, first of all, neurological diseases and mental disorders [131].

**Figure 8:**
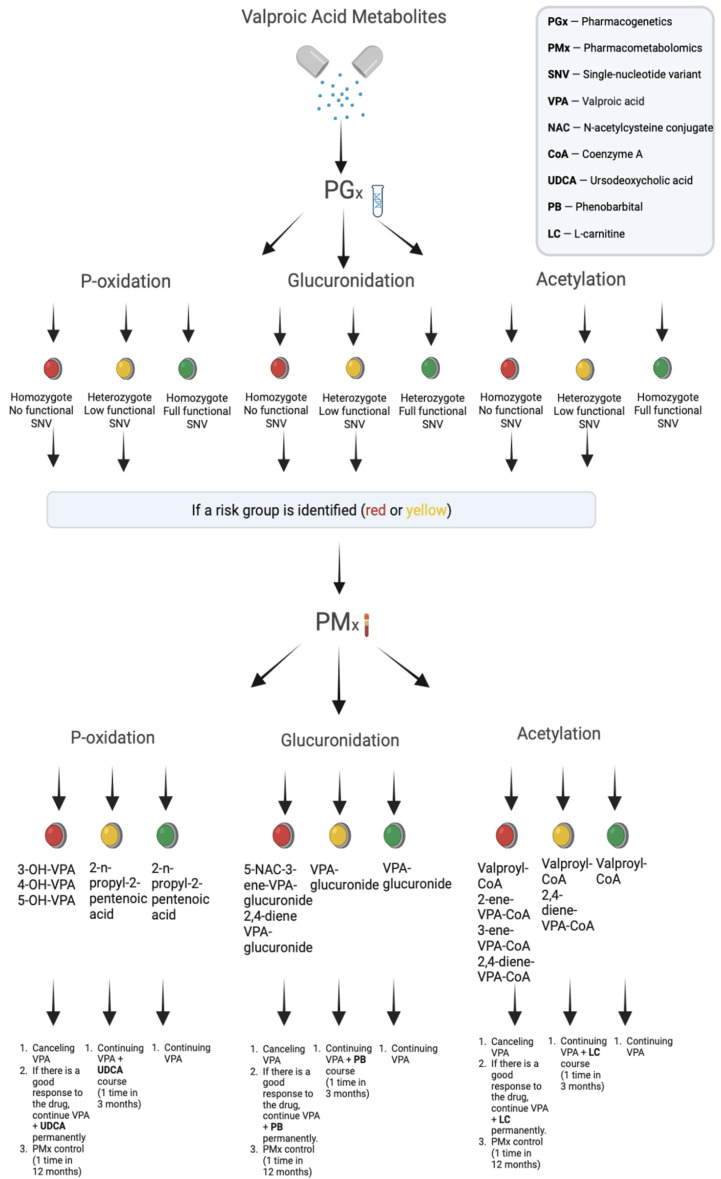
«The pharmacogenetics-informed pharmacometabolomics» approach to assessing the risk of development and correction of adverse drug reactions induced by valproic acid metabolites.

The pharmacogenomics and pharmacometabolomics of VPA can be particularly useful when used together (Figure 8).

The genome is consistent with various cell and tissue types in humans, although epigenomics and the regulation of genomic expression are highly dependent on cells and tissues, and since pharmacometabolomics is an “-omic” type of data that appears to be most closely associated with clinical phenotypes [128,132], the integration of pharmacogenomics and pharmacometabolomics may be particularly useful in achieving an optimal balance between the efficacy and safety of VPA. The use of genomic methods, such as genome-wide association studies, has been very successful [132], especially in situations where the phenotype of VPA drug metabolism in the liver in a particular patient is clearly defined (for example, P-oxidation involving cytochrome P450 isoenzymes) [37]. However, the use of pharmacogenomic methods alone was less successful when applied to complex and less defined phenotypes (for example, the «P-oxidation + glucuronidation + acetylation») of VPA metabolism in patients with common and rare neurological diseases and mental disorders. It is for this reason that the use of our proposed personalized research strategy, which starts with VPA pharmacogenomics and allows genomic data to “guide” the VPA pharmacometabolomics study, can be particularly useful in selected clinical situations.

For example, with a genetically determined increase in the level of toxic metabolites associated with impaired mitochondrial oxidation, in which VPA inhibits carnitine biosynthesis by reducing the concentration of alpha-ketoglutarate and can contribute to the development of carnitine deficiency, L-carnitine preparations are the drug of choice for the correction of VPA-induced ADRs. It is known that L-carnitine is an amino acid derivative that is an important cofactor in the beta-oxidation of fatty acids [117], including VPA. L-carnitine is increasingly recommended for the treatment of hyperammonemia caused by VPA. It is a corrective drug for VPA-induced hyperammonemia and other ADRs of this drug and acts as an acceptor of toxic metabolites such as valproil-CoA. Valproil-carnitine ester is subsequently excreted in the urine. As a result of the normalization of the ratio of acyl-CoA/CoA-OH in mitochondria, the reverse inhibition of β-oxidation occurs, which, in turn, leads to the formation of acetyl-CoA. The formation of acetyl-CoA allows the formation of NAGA and the subsequent detoxification of ammonia to urea, thereby correcting hyperammonemia. L-carnitine supplementation is well tolerated, and reports of possible ADRs are rare [38,133].

With an increase in the level of toxic metabolites caused by a violation of P-oxidation of VPA, in order to correct VPA-induced ADRs, it is recommended to prescribe ursodeoxycholic acid (UDCA) drugs. It is known that UDCA is a primary bile acid that is easily synthesized in the human body [134]. In addition, UDCA has a direct cytoprotective effect due to the stabilization of the hepatocyte membrane and improvement of mitochondrial oxidative phosphorylation [135]. In addition, UDCA prevents the transition of the mitochondrial membrane permeability and promotes a decrease in the VPA-induced high serum levels of alanine aminotransferase and aspartate aminotransferase [136]. An immunomodulatory effect was found in UDCA; its mechanism is explained by a decrease in the expression of HLA class I proteins in hepatocytes and the anti-apoptotic effect of UDCA due to a decrease in the production of reactive oxygen species [137].

With an increase in the level of metabolites associated with impaired glucuronidation [138] of VPA drugs, phenobarbital is a well-established corrector of VPA-induced ADRs [139]. Phenobarbital has been used for many years for detoxification in patients suffering from various forms of sedative dependence, as well as intoxication with psychotropic drugs. There is also a reliable clinical test using pentobarbital to assess the tolerance of psychotropic drugs and thus determine the dosing regimen to replace phenobarbital [140].

Thus, the presented personalized approach to the cumulative risk assessment for the development and correction of ADRs associated with an increase in the level of toxic VPA metabolites in the blood and urine allows clinicians to adapt previously known classical methods of treatment using valproate to individual patients from the standpoint of pharmacogenomics and pharmacometabolomics. It should be recognized that practicing neurologists and psychiatrists often lack adequate objective data to truly personalize psychopharmacotherapy [141]. In the future, a new era of personalized medicine may enable the effective use of a combination of pharmacogenomics and pharmacometabolomics to identify major molecular pathways and genetic variations in candidate genes encoding key VPA biotransformation enzymes. This may help us explain the individual phenotypes of patients with neurological disease and psychiatric disorders and ensure that an optimal balance is achieved between the efficacy and safety of valproates.

So, the term “pharmacometabolomics-informed pharmacogenomics”, first proposed 10 years ago (in 2012) [142], represents one promising strategy at the intersection of pharmacometabolomics and pharmacogenomics and gives us the key to understanding the mechanisms of formation, action, and utilization of VPA metabolites. The alternative approach proposed by us, “pharmacogenetics-informed pharmacometabolomics”, makes it possible to individually predict the risk of elevated levels of toxic VPA metabolites based on lifelong unchanged PGx results that do not change throughout a person’s life and optimize the choice and scope of metabolic studies (in particular, gas-liquid chromatography/mass spectrometry). This is an important aspect of the movement towards 4P medicine (prediction, prevention, personalization, and participation) [143]. In addition, it is promising and important to compare the results of pharmacogenomic and pharmacometabolic studies of therapeutic and toxic metabolites of VPA in the future in order to identify genetic variants that contribute to metabolomic variation but are not dependent on drug response to valproate.

## 9. Limitations

Within the limits of restrictions, we would like to note that the search was carried out only in PubMed, Web of Science, Springer, Google Scholar, and e-Library databases. We have focused on the well-known and potential role of therapeutic and toxic metabolites in the efficacy and safety of VPA in this narrative review, but we have not analyzed clinical randomized trials of the efficacy of valproates, including VPA and its salts. It is possible that the level of therapeutic and toxic metabolites of VPA in human biological fluids was also studied in some of these trails. Undoubtedly, it would be interesting to prepare a systematic review in the future.

## 10. Conclusions

Valproates are psychotropic drugs that are widely used for neurological diseases and mental disorders. In addition, the indications for the appointment of valproate have been expanding in recent years in connection with the study of the mechanism of action of therapeutic and toxic metabolites of VPA in the human body. The most studied pathways of VPA metabolism include: β-oxidation in the tricarboxylic acid cycle (acetylation); oxidation involving cytochrome P-450 isoenzymes in the liver (P-oxidation); and glucuronidation. The complex metabolism of VPA explains the diversity of its active and inactive metabolites, which have therapeutic, neutral, or toxic effects. It is known that some active metabolites of VPA may have a stronger clinical effect than VPA itself. In future studies of VPA metabolites, it is important to use pharmacometabolomics together with pharmacogenomics to improve understanding of the biochemical pathways for the therapeutic and toxic metabolites formation, which may play an important role in individual variations in the phenotypes of the expected drug response to VPA, as well as in individual variations in the metabotypes associated with the risk of developing VPA-induced ADRs. An illustration of the importance of this approach is the proposed personalized approach to the cumulative assessment of the risk of developing VPA-induced ADRs and the choice of tactics for their correction, depending on the pharmacogenetic profile of the patient and the level of therapeutic and toxic VPA metabolites in human body fluids (blood, urine), and possibly saliva.

## Figures and Tables

**Table 1:** Serum and Plasma Therapeutic Metabolites of Valproic Acid.

Metabolite	HMDB Number	Clinical Effect	References
VPA-glucuronide	0000901	Therapeutic Neutral	[18,98]
2-n-propyl-2-pentenoic acid	0013902	Therapeutic	[99]
Valproyl-CoA	0013115	Therapeutic	[100]
4-ene-VPA	0013897	Therapeutic	[101]
2-ene-VPA	0013902	Therapeutic	[101]

Note: CoA–coenzyme A; HMDB–The Human Metabolome Database [https://hmdb.ca/ (accessed on 12 November 2022)]; VPA–valproic acid.

**Table 2:** Urinary Therapeutic Metabolites of Valproic Acid.

Metabolite	HMDB Number	Clinical Effect	References
VPA-glucuronide	0000901	Therapeutic Neutral	[18,98]
2-n-propyl-2-pentenoic acid	0013902	Therapeutic	[99]
4-ene-VPA	0013897	Therapeutic	[100]
2-ene-VPA	0013902	Therapeutic	[101]

Note: CoA–coenzyme A; HMDB–The Human Metabolome Database [https://hmdb.ca/ (accessed on 12 November 2022)]; VPA–valproic acid.

**Table 3:** Serum and Plasma Toxic Metabolites of Valproic Acid.

Metabolite	HMDB Number	Clinical Effect	References
3-hydroxy valproic acid	0013899	Toxic	[95]
4-hydroxy valproic acid	0013900	Toxic	[112]
5-hydroxy valproic acid	0013898	Toxic	[95]
Valproyl Coenzyme A	0013115	Toxic	[95]
Valproylcarnitine	0259757	Toxic	[113]
2-n-propyl-2-pentenoic acid	0013902	Toxic	[114]
2-n-propyl-4-oxopentanoic acid	0060683	Toxic	[114]
2-Propyl-2,4-pentadienoic acid	0060682	Toxic	[23]
3-oxovalproic acid	0060685	Toxic	[95]
4-ene-valproic acid	0013897	Toxic	[95]
2-ene-valproic acid	0013902	Toxic	[114]
2-ene-valproic acid Coenzyme A	0060714	Toxic	[38]
3-ene-valproic acid Coenzyme A	0060740	Toxic	[95]
2-diene-valproic acid glucuronide		Toxic	[23]

Note: HMDB–The Human Metabolome Database [https://hmdb.ca/ (accessed on 12 November 2022)].

**Table 4:** Urinary Toxic Metabolites of Valproic Acid.

Metabolite	HMDB Number	Clinical Effect	References
3-hydroxy valproic acid	0013899	Toxic	[95]
4-hydroxy valproic acid	0013900	Toxic	[112]
5-hydroxy valproic acid	0013898	Toxic	[95]
3-oxovalproic acid	0060685	Toxic	[95]
2-ene-valproic acid	0013902	Toxic	[114]
2-ene-valproic acid Coenzyme A	0060714	Toxic	[38]
3-ene-valproic acid Coenzyme A	0060740	Toxic	[95]
2-propyl-2,4-pentadienoic acid	0060682	Toxic	[23]
2-n-propyl-4-oxopentanoic acid	0060683	Toxic	[114]

Note: HMDB–The Human Metabolome Database [https://hmdb.ca/ (accessed on 12 November 2022)].

## Appendix A

**Table A1:** Genetic Biomarkers of Valproic Acid Metabolism Retardation.

Gene (OMIM Number)	Chromosomal Location	Protein	SNV (RSID)	References
			Oxidation	
*CYP2A6* (122720)	19q13.2	Isoenzyme 2A6 of cytochrome P450	*2 (rs1801272)	The homozygous AA genotype—a biomarker for a high risk of impaired VPA oxidation and increased levels of toxic metabolites.
			*5 (rs5031017)	The homozygous TT genotype—a biomarker for a high risk of impaired VPA oxidation and increased levels of toxic metabolites. The heterozygous GT genotype—a biomarker for a high risk of impaired VPA oxidation and increased levels of toxic metabolites.
			rs (111033610)	The homozygous CC genotype—a biomarker for a high risk of impaired VPA oxidation and increased levels of toxic metabolites.
			rs28399447	The homozygous CC genotype—a biomarker for a high risk of impaired VPA oxidation and increased levels of toxic metabolites.
			*6 (rs4986891)	The homozygous AA genotype—a biomarker for a high risk of impaired VPA oxidation and increased levels of toxic metabolites.
			*7 (rs5031016)	The homozygous CC genotype—a biomarker for a high risk of impaired VPA oxidation and increased levels of toxic metabolites.
			*9A (s28399433)	The homozygous GG genotype—a biomarker for a high risk of impaired VPA oxidation and increased levels of toxic metabolites.
			*11 (rs28399447)	The homozygous CC genotype—a biomarker for a high risk of impaired VPA oxidation and increased levels of toxic metabolites.
			*17 (rs28399454)	The homozygous AA genotype—a biomarker for a high risk of impaired VPA oxidation and increased levels of toxic metabolites.
			*18 (rs1809810)	The homozygous TT genotype—a biomarker for a high risk of impaired VPA oxidation and increased levels of toxic metabolites.
			*19 (rs1809810 )	The homozygous TT genotype—a biomarker for a high risk of impaired VPA oxidation and increased levels of toxic metabolites.
			*23 (rs56256500)	The homozygous TT genotype—a biomarker for a high risk of impaired VPA oxidation and increased levels of toxic metabolites.
			*24A (rs143731390)	The homozygous TT genotype—a biomarker for a high risk of impaired VPA oxidation and increased levels of toxic metabolites.
			*24A (rs72549435)	The homozygous TT genotype—a biomarker for a high risk of impaired VPA oxidation and increased levels of toxic metabolites.
			*26 (rs59552350)	The homozygous GG genotype—a biomarker for a high risk of impaired VPA oxidation and increased levels of toxic metabolites.
			*26 (rs4986891)	The homozygous TT genotype—a biomarker for a high risk of impaired VPA oxidation and increased levels of toxic metabolites.
			*27 (rs2839944)	The homozygous CC genotype—a biomarker for a high risk of impaired VPA oxidation and increased levels of toxic metabolites.
			*27 (rs28399445)	The homozygous AA genotype—a biomarker for a high risk of impaired VPA oxidation and increased levels of toxic metabolites.
			*35 (rs143731390)	The homozygous TT genotype—a biomarker for a high risk of impaired VPA oxidation and increased levels of toxic metabolites.
			*41 (rs140471703 7)	The homozygous AA genotype—a biomarker for a high risk of impaired VPA oxidation and increased levels of toxic metabolites.
*CYP2C9* (601130)	10q23.33	Isoenzyme 2A6 of cytochrome P450	*2 (rs1799853)	The homozygous TT genotype—a biomarker for a high risk of impaired VPA oxidation and increased levels of toxic metabolites.
			*3 (rs1057910)	The homozygous CC genotype—a biomarker for a high risk of impaired VPA oxidation and increased levels of toxic metabolites.
			*4 (rs56165452)	The homozygous AA genotype—a biomarker for a high risk of impaired VPA oxidation and increased levels of toxic metabolites.
			*5 (rs28371686)	The homozygous GG genotype– a biomarker for a high risk of impaired VPA oxidation and increased levels of toxic metabolites.
			*8 (rs7900194)	The homozygous AA genotype—a biomarker for a high risk of impaired VPA oxidation and increased levels of toxic metabolites.
			*11 (rs28371685)	The homozygous TT genotype—a biomarker for a high risk of impaired VPA oxidation and increased levels of toxic metabolites.
			*13 (rs72558187)	The homozygous CC genotype—a biomarker for a high risk of impaired VPA oxidation and increased levels of toxic metabolites.
			*15 (rs72558190)	The homozygous AA genotype—a biomarker for a high risk of impaired VPA oxidation and increased levels of toxic metabolites.
*CYP2B6* (123930)	19q13.2	Isoenzyme 2A6 of cytochrome P450	*5A (rs3211371)	The homozygous AA genotype—a biomarker for a high risk of impaired VPA oxidation and increased levels of toxic metabolites.
			*8 (rs12721655)	The homozygous GG genotype—a biomarker for a high risk of impaired VPA oxidation and increased levels of toxic metabolites.
			*18 (rs28399499)	The homozygous AA genotype—a biomarker for a high risk of impaired VPA oxidation and increased levels of toxic metabolites.
			*27 (rs36079186)	The homozygous CC genotype—a biomarker for a high risk of impaired VPA oxidation and increased levels of toxic metabolites.
			*28 (rs34097093)	The homozygous TT genotype—a biomarker for a high risk of impaired VPA oxidation and increased levels of toxic metabolites.
*CYP3A4* (124010)	7q22.1	Isoenzyme 2A6 of cytochrome P450	*3 (rs4986910)	The homozygous CC genotype—a biomarker for a high risk of impaired VPA oxidation and increased levels of toxic metabolites.
			*17 (rs4987161)	The homozygous CC genotype—a biomarker for a high risk of impaired VPA oxidation and increased levels of toxic metabolites.
			*18 (rs28371759)	The homozygous CC genotype—a biomarker for a high risk of impaired VPA oxidation and increased levels of toxic metabolites.
			*20 (rs67666821)	The homozygous TT genotype—a biomarker for a high risk of impaired VPA oxidation and increased levels of toxic metabolites.
*CYP2C19* (124020)	10q23.33	Isoenzyme 2A6 of cytochrome P450	*2 (rs4244285)	The homozygous AA genotype—a biomarker for a high risk of impaired VPA oxidation and increased levels of toxic metabolites.
			*4 (rs28399504)	The homozygous GG genotype—a biomarker for a high risk of impaired VPA oxidation and increased levels of toxic metabolites.
			*5 (rs56337013)	The homozygous TT genotype—a biomarker for a high risk of impaired VPA oxidation and increased levels of toxic metabolites.
			*8 (rs41291556)	The homozygous CC genotype—a biomarker for a high risk of impaired VPA oxidation and increased levels of toxic metabolites.
*CYP2D6* (124030)	22q13.2	Isoenzyme 2A6 of cytochrome P450	*4 (rs3892097)	The homozygous AA genotype—a biomarker for a high risk of impaired VPA oxidation and increased levels of toxic metabolites.
			*4F (rs3892097)	The homozygous AA genotype—a biomarker for a high risk of impaired VPA oxidation and increased levels of toxic metabolites.
			*4G (rs3892097)	The homozygous AA genotype—a biomarker for a high risk of impaired VPA oxidation and increased levels of toxic metabolites.
			*4H (rs3892097)	The homozygous AA genotype—a biomarker for a high risk of impaired VPA oxidation and increased levels of toxic metabolites.
			*8 (rs5030865)	The homozygous AA genotype—a biomarker for a high risk of impaired VPA oxidation and increased levels of toxic metabolites.
			*10 (rs1065852)	The homozygous TT genotype—a biomarker for a high risk of impaired VPA oxidation and increased levels of toxic metabolites.
			*17 (rs28371706)	The homozygous TT genotype—a biomarker for a high risk of impaired VPA oxidation and increased levels of toxic metabolites.
			Glucuronization	
*UGT1A1* (191740)	2q37.1	UDP-glucuronosyltransferase	rs111033539	The homozygous TT genotype (Crigler Najjar syndrome)—a biomarker for a high risk of impaired VPA glucuronization and increased levels of toxic metabolites.
			rs111033540	The homozygous GG genotype (Crigler Najjar syndrome, Gilbert’s syndrome)—a biomarker for a high risk of impaired VPA glucuronization and increased levels of toxic metabolites.
			rs111033541	The homozygous GG genotype (Crigler Najjar syndrome)—a biomarker for a high risk of impaired VPA glucuronization and increased levels of toxic metabolites.
*UGT1A3* (606428)	2q37.1	UDP-glucuronosyltransferase	rs111033541	The homozygous GG genotype (Crigler Najjar syndrome)—a biomarker for a high risk of impaired VPA glucuronization and increased levels of toxic metabolites.
			rs10929302	A biomarker for a high risk of impaired VPA glucuronization and increased levels of toxic metabolites.
			rs111033539	The homozygous TT genotype (Crigler Najjar syndrome)—a biomarker for a high risk of impaired VPA glucuronization and increased levels of toxic metabolites.
191740			rs111033540	The homozygous GG genotype (Crigler-Najjar syndrome, Gilbert’s syndrome)—a biomarker for a high risk of impaired VPA glucuronization and increased levels of toxic metabolites.
			rs11891311	Allele G—a biomarker for a high risk of impaired VPA glucuronization and increased levels of toxic metabolites (встрeчaeтся чащe у азиатoв).
			rs281865418	The homozygous GG; AA genotypes (Crigler-Najjar syndrome)—a biomarker for a high risk of impaired VPA glucuronization and increased levels of toxic metabolites.
			rs28934877	The homozygous GG; CC genotypes (Crigler-Najjar syndrome Gilbert’s syndrome)—a biomarker for a high risk of impaired VPA glucuronization and increased levels of toxic metabolites.
			rs3064744	The homozygous TA; TA genotypes—a biomarker for a high risk of impaired VPA glucuronization and increased levels of toxic metabolites.
			rs35003977	The homozygous GG genotype (Hyperbilirubinemia Gilbert’s syndrome; Crigler-Najjar syndrome)—a biomarker for a high risk of impaired VPA glucuronization and increased levels of toxic metabolites.
			rs34993780	The homozygous AA; CC; GG genotypes (Lucey-Driscoll syndrome; Crigler-Najjar syndrome; Hyperbilirubinemia)—a biomarker for a high risk of impaired VPA glucuronization and increased levels of toxic metabolites.
			rs35350960	The homozygous AA; TT genotypes—a biomarker for a high risk of impaired VPA glucuronization and increased levels of toxic metabolites.
			rs3755319	The homozygous GG genotype (Lucey-Driscoll syndrome)—a biomarker for a high risk of impaired VPA glucuronization and increased levels of toxic metabolites.
			rs386576623	The homozygous GG genotype (Crigler-Najjar syndrome; Gilbert’s syndrome)—a biomarker for a high risk of impaired VPA glucuronization and increased levels of toxic metabolites.
			rs4124874	The homozygous CC genotype (Gilbert syndrome)—a biomarker for a high risk of impaired VPA glucuronization and increased levels of toxic metabolites.
			rs4148323	The homozygous AA genotype—a biomarker for a high risk of impaired VPA glucuronization and increased levels of toxic metabolites.
			rs587776761	The homozygous AA genotype (Gilbert syndrome) —a biomarker for a high risk of impaired VPA glucuronization and increased levels of toxic metabolites.
			rs587776763	The homozygous TT genotype (Gilbert syndrome) —a biomarker for a high risk of impaired VPA glucuronization and increased levels of toxic metabolites.
			rs587776764	The homozygous CC genotype (Gilbert syndrome) —a biomarker for a high risk of impaired VPA glucuronization and increased levels of toxic metabolites.
			rs587776765	The homozygous TT genotype (Crigler-Najjar syndrome) —a biomarker for a high risk of impaired VPA glucuronization and increased levels of toxic metabolites.
			rs587776766	The homozygous GG genotype (Crigler-Najjar syndrome)—a biomarker for a high risk of impaired VPA glucuronization and increased levels of toxic metabolites.
			rs587784535	The homozygous TT genotype (Hyperbilirubinemia) —a biomarker for a high risk of impaired VPA glucuronization and increased levels of toxic metabolites.
			rs62625011	The homozygous AA genotype (Crigler-Najjar syndrome)—a biomarker for a high risk of impaired VPA glucuronization and increased levels of toxic metabolites.
			rs6742078	The homozygous TT genotype (16% bilirubin levels increased risk of gallstones) —a biomarker for a high risk of impaired VPA glucuronization and increased levels of toxic metabolites.
			rs72551341	The homozygous AA genotype (Crigler-Najjar syndrome)—a biomarker for a high risk of impaired VPA glucuronization and increased levels of toxic metabolites.
			rs72551348	The homozygous GG genotype (Crigler-Najjar syndrome)—a biomarker for a high risk of impaired VPA glucuronization and increased levels of toxic metabolites.
			rs72551349	The homozygous TT genotype (Crigler Najjar syndrome Gilbert’s syndrome) —a biomarker for a high risk of impaired VPA glucuronization and increased levels of toxic metabolites.
			rs72551350	The homozygous TT genotype (Crigler-Najjar syndrome)—a biomarker for a high risk of impaired VPA glucuronization and increased levels of toxic metabolites.
			rs72551351	The homozygous GG genotype (Crigler-Najjar syndrome)—a biomarker for a high risk of impaired VPA glucuronization and increased levels of toxic metabolites.
			rs72551353	The homozygous TT genotype (Crigler-Najjar syndrome)—a biomarker for a high risk of impaired VPA glucuronization and increased levels of toxic metabolites.
			rs755218546	The homozygous AA genotype—a biomarker for a high risk of impaired VPA glucuronization and increased levels of toxic metabolites.
			rs886039771	The homozygous AA genotype (Crigler-Najjar syndrome)—a biomarker for a high risk of impaired VPA glucuronization and increased levels of toxic metabolites.
*UGT1A4* (606429)	2q37.1	UDP-glucuronosyltransferase	rs111033539	The homozygous TT genotype (Crigler-Najjar syndrome) —a biomarker for a high risk of impaired VPA glucuronization and increased levels of toxic metabolites.
			rs111033540	The homozygous AA genotype (Crigler-Najjar syndrome; Gilbert’s syndrome)—a biomarker for a high risk of impaired VPA glucuronization and increased levels of toxic metabolites.
			rs111033541	The homozygous GG genotype (Crigler-Najjar syndrome)—a biomarker for a high risk of impaired VPA glucuronization and increased levels of toxic metabolites.
			rs2011425	The homozygous GG; AA genotypes—a biomarker for a high risk of impaired VPA glucuronization and increased levels of toxic metabolites.
			rs281865418	The homozygous GG; AA genotypes (Crigler-Najjar syndrome) —a biomarker for a high risk of impaired VPA glucuronization and increased levels of toxic metabolites.
			rs28934877	The homozygous GG; CC genotypes (Crigler-Najjar syndrome; Gilbert’s syndrome) —a biomarker for a high risk of impaired VPA glucuronization and increased levels of toxic metabolites.
			rs34993780	The homozygous GG; AA; CC genotypes (Lucey-Driscoll syndrome Crigler-Najjar syndrome Hyperbilirubinemia) —a biomarker for a high risk of impaired VPA glucuronization and increased levels of toxic metabolites.
			rs35003977	The homozygous GG genotype (Hyperbilirubinemia Gilbert’s syndrome; Crigler-Najjar syndrome) —a biomarker for a high risk of impaired VPA glucuronization and increased levels of toxic metabolites.
			rs3755319	The homozygous GG genotype (Lucey-Driscoll syndrome)—a biomarker for a high risk of impaired VPA glucuronization and increased levels of toxic metabolites.
			rs386576623	The homozygous GG genotype (Crigler-Najjar syndrome Gilbert’s syndrome)—a biomarker for a high risk of impaired VPA glucuronization and increased levels of toxic metabolites.
			rs4124874	The homozygous AA genotype (Crigler Najjar syndrome)—a biomarker for a high risk of impaired VPA glucuronization and increased levels of toxic metabolites.
			rs587776761	The homozygous AA genotype (Crigler Najjar syndrome)—a biomarker for a high risk of impaired VPA glucuronization and increased levels of toxic metabolites.
			rs587776763	The homozygous TT genotype (Crigler Najjar syndrome)—a biomarker for a high risk of impaired VPA glucuronization and increased levels of toxic metabolites.
			rs587776764	The homozygous CC genotype (Crigler Najjar syndrome)—a biomarker for a high risk of impaired VPA glucuronization and increased levels of toxic metabolites.
			rs587776765	The homozygous TT genotype (Crigler Najjar syndrome)—a biomarker for a high risk of impaired VPA glucuronization and increased levels of toxic metabolites.
			rs587776766	The homozygous GG genotype (Crigler Najjar syndrome)—a biomarker for a high risk of impaired VPA glucuronization and increased levels of toxic metabolites.
			rs587784535	The homozygous TT genotype (Hyperbilirubinemia) —a biomarker for a high risk of impaired VPA glucuronization and increased levels of toxic metabolites.
			rs62625011	The homozygous AA genotype (Crigler Najjar syndrome)—a biomarker for a high risk of impaired VPA glucuronization and increased levels of toxic metabolites.
			rs6742078	The homozygous TT genotype (+16% bilirubin levels increased risk of gallstones) —a biomarker for a high risk of impaired VPA glucuronization and increased levels of toxic metabolites.
			rs72551341	The homozygous AA genotype (Crigler-Najjar syndrome)—a biomarker for a high risk of impaired VPA glucuronization and increased levels of toxic metabolites.
			rs72551348	The homozygous GG genotype (Crigler-Najjar syndrome)—a biomarker for a high risk of impaired VPA glucuronization and increased levels of toxic metabolites.
			rs72551349	The homozygous TT genotype (Crigler Najjar syndrome Gilbert’s syndrome) —a biomarker for a high risk of impaired VPA glucuronization and increased levels of toxic metabolites.
			rs72551350	The homozygous TT genotype (Crigler Najjar syndrome)—a biomarker for a high risk of impaired VPA glucuronization and increased levels of toxic metabolites.
			rs72551351	The homozygous TT genotype (Crigler Najjar syndrome)—a biomarker for a high risk of impaired VPA glucuronization and increased levels of toxic metabolites.
			rs72551353	The homozygous TT genotype (Crigler Najjar syndrome)—a biomarker for a high risk of impaired VPA glucuronization and increased levels of toxic metabolites.
			rs755218546	The homozygous AA genotype —a biomarker for a high risk of impaired VPA glucuronization and increased levels of toxic metabolites.
			rs797046090	Genotypes CGTA;CGTA (Hyperbilirubinemia) —a biomarker for a high risk of impaired VPA glucuronization and increased levels of toxic metabolites.
			rs886039771	The homozygous AA genotype (Crigler-Najjar syndrome)—a biomarker for a high risk of impaired VPA glucuronization and increased levels of toxic metabolites.
*UGT1A6* (606431)	2q37.1	UDP-glucuronosyltransferase	rs111033539	The homozygous TT genotype (Crigler-Najjar syndrome)—a biomarker for a high risk of impaired VPA glucuronization and increased levels of toxic metabolites.
			rs111033540	The homozygous GG genotype (Crigler-Najjar syndrome Gilbert’s syndrome)—a biomarker for a high risk of impaired VPA glucuronization and increased levels of toxic metabolites.
			rs111033541	The homozygous GG genotype (Crigler-Najjar syndrome)—a biomarker for a high risk of impaired VPA glucuronization and increased levels of toxic metabolites.
			rs2011425	The homozygous AA; GG genotypes—a biomarker for a high risk of impaired VPA glucuronization and increased levels of toxic metabolites.
			rs281865418	The homozygous AA; GG genotypes (Crigler Najjar syndrome)—a biomarker for a high risk of impaired VPA glucuronization and increased levels of toxic metabolites.
			rs28934877	The homozygous CC; GG genotypes (Crigler Najjar syndrome)—a biomarker for a high risk of impaired VPA glucuronization and increased levels of toxic metabolites.
			rs34993780	The homozygous AA; GG genotypes (Lucey-Driscoll syndrome; Crigler-Najjar syndrome Hyperbilirubinemia)—a biomarker for a high risk of impaired VPA glucuronization and increased levels of toxic metabolites.
			rs35003977	The homozygous GG genotype (Hyperbilirubinemia Gilbert’s syndrome; Crigler-Najjar syndrome)—a biomarker for a high risk of impaired VPA glucuronization and increased levels of toxic metabolites.
			rs3755319	The homozygous GG genotype (Lucey-Driscoll syndrome)—a biomarker for a high risk of impaired VPA glucuronization and increased levels of toxic metabolites.
			rs386576623	The homozygous GG genotype (Crigler-Najjar syndrome; Gilbert’s syndrome)—a biomarker for a high risk of impaired VPA glucuronization and increased levels of toxic metabolites.
			rs4124874	The homozygous GG genotype (Gilbert’s syndrome)—a biomarker for a high risk of impaired VPA glucuronization and increased levels of toxic metabolites.
			rs587776761	The homozygous AA genotype (Crigler Najjar syndrome)—a biomarker for a high risk of impaired VPA glucuronization and increased levels of toxic metabolites.
			rs587776763	The homozygous TT genotype (Crigler Najjar syndrome)—a biomarker for a high risk of impaired VPA glucuronization and increased levels of toxic metabolites.
			rs587776764	The homozygous CC genotype (Crigler Najjar syndrome)—a biomarker for a high risk of impaired VPA glucuronization and increased levels of toxic metabolites.
			rs587776765	The homozygous TT genotype (Crigler Najjar syndrome)—a biomarker for a high risk of impaired VPA glucuronization and increased levels of toxic metabolites.
			rs587776766	The homozygous GG genotype (Crigler Najjar syndrome)—a biomarker for a high risk of impaired VPA glucuronization and increased levels of toxic metabolites.
			rs587784535	The homozygous TT genotype (Hyperbilirubinemia)—a biomarker for a high risk of impaired VPA glucuronization and increased levels of toxic metabolites.
			rs62625011	The homozygous AA genotype (Crigler Najjar syndrome)—a biomarker for a high risk of impaired VPA glucuronization and increased levels of toxic metabolites.
			rs6742078	The homozygous TT genotype (+16% bilirubin levels increased risk of gallstones) —a biomarker for a high risk of impaired VPA glucuronization and increased levels of toxic metabolites.
			rs72551341	The homozygous AA genotype (Crigler Najjar syndrome)—a biomarker for a high risk of impaired VPA glucuronization and increased levels of toxic metabolites.
			rs72551348	The homozygous GG genotype (Crigler Najjar syndrome)—a biomarker for a high risk of impaired VPA glucuronization and increased levels of toxic metabolites.
*UGT1A8* (606433)	2q37.1	UDP-glucuronosyltransferase	rs111033539	The homozygous TT genotype (Crigler Najjar syndrome)—a biomarker for a high risk of impaired VPA glucuronization and increased levels of toxic metabolites.
			rs111033540	The homozygous GG genotype (Crigler-Najjar syndrome; Gilbert’s syndrome)—a biomarker for a high risk of impaired VPA glucuronization and increased levels of toxic metabolites.
			rs111033541	The homozygous GG genotype (Crigler Najjar syndrome)—a biomarker for a high risk of impaired VPA glucuronization and increased levels of toxic metabolites.
			rs2011425	The homozygous GG; AA genotypes—a biomarker for a high risk of impaired VPA glucuronization and increased levels of toxic metabolites.
			rs281865418	The homozygous GG; AA genotypes (Crigler Najjar syndrome)—a biomarker for a high risk of impaired VPA glucuronization and increased levels of toxic metabolites.
			rs28934877	The homozygous GG; CC genotypes (Crigler-Najjar syndrome; Gilbert’s syndrome)—a biomarker for a high risk of impaired VPA glucuronization and increased levels of toxic metabolites.
			rs34993780	The homozygous AA; GG; CC genotypes (Lucey-Driscoll syndrome; Crigler-Najjar syndrome Hyperbilirubinemia)—a biomarker for a high risk of impaired VPA glucuronization and increased levels of toxic metabolites.
			rs35003977	The homozygous GG genotype (Hyperbilirubinemia Gilbert’s syndrome; Crigler-Najjar syndrome)—a biomarker for a high risk of impaired VPA glucuronization and increased levels of toxic metabolites.
			rs3755319	The homozygous GG genotype (Lucey-Driscoll syndrome)—a biomarker for a high risk of impaired VPA glucuronization and increased levels of toxic metabolites.
			rs386576623	The homozygous GG genotype (Crigler-Najjar syndrome; Gilbert’s syndrome)—a biomarker for a high risk of impaired VPA glucuronization and increased levels of toxic metabolites.
			rs4124874	The homozygous CC genotype (Gilbert syndrome)—a biomarker for a high risk of impaired VPA glucuronization and increased levels of toxic metabolites.
			rs587776761	The homozygous AA genotype (Crigler Najjar syndrome)—a biomarker for a high risk of impaired VPA glucuronization and increased levels of toxic metabolites.
*UGT1A9* (606434)	2q37.1	UDP-glucuronosyltransferase	rs111033539	The homozygous TT genotype (Crigler Najjar syndrome)—a biomarker for a high risk of impaired VPA glucuronization and increased levels of toxic metabolites.
			rs111033540	The homozygous GG genotype (Crigler-Najjar syndrome; Gilbert’s syndrome)—a biomarker for a high risk of impaired VPA glucuronization and increased levels of toxic metabolites.
			rs111033541	The homozygous GG genotype (Crigler-Najjar syndrome)—a biomarker for a high risk of impaired VPA glucuronization and increased levels of toxic metabolites.
*UGT1A10* (606435)	2q37.1	UDP-glucuronosyltransferase	rs72551350	The homozygous TT genotype (Crigler Najjar syndrome)—a biomarker for a high risk of impaired VPA glucuronization and increased levels of toxic metabolites.
			rs17863783	The homozygous TT genotype—a biomarker for a high risk of impaired VPA glucuronization and increased levels of toxic metabolites.
*UGT2B7* (600068)	4q13.2	UDP-glucuronosyltransferase	rs12233719	The homozygous TT genotype —a biomarker for a high risk of impaired VPA glucuronization and increased levels of toxic metabolites.
			rs7438135	The homozygous GG genotype—a biomarker for a high risk of impaired VPA glucuronization and increased levels of toxic metabolites.
			rs7439366	The homozygous TT genotype—a biomarker for a high risk of impaired VPA glucuronization and increased levels of toxic metabolites.
			rs7668258	The homozygous TT genotype—a biomarker for a high risk of impaired VPA glucuronization and increased levels of toxic metabolites.
*UGT2B15* (600069)	4q13.2	UDP-glucuronosyltransferase	rs1902023	The homozygous GG genotype—a biomarker for a high risk of impaired VPA glucuronization and increased levels of toxic metabolites.
			Acetylation	
*ACADSB* (600301)	10q26.13	Acyl-CoA Dehydrogenase	rs58639322	The homozygous TT genotype —a biomarker for a high risk of impaired VPA acetylation and increased levels of toxic metabolites.
			rs142095937	The homozygous GG genotype —a biomarker for a high risk of impaired VPA acetylation and increased levels of toxic metabolites.
			rs137852649	The homozygous TT genotype—a biomarker for a high risk of impaired VPA acetylation and increased levels of toxic metabolites.
			rs760791287	The homozygous TT genotype—a biomarker for a high risk of impaired VPA acetylation and increased levels of toxic metabolites.
			rs147936696	The homozygous AA genotype —a biomarker for a high risk of impaired VPA acetylation and increased levels of toxic metabolites.
			rs387906409	The homozygous AA genotype—a biomarker for a high risk of impaired VPA acetylation and increased levels of toxic metabolites.
*ACSM1* (614357)	16p12.3	Acyl-CoA Synthetase 1	rs163234	The homozygous AA genotype —a biomarker for a high risk of impaired VPA acetylation and increased levels of toxic metabolites.
			rs151222	The homozygous CC genotype —a biomarker for a high risk of impaired VPA acetylation and increased levels of toxic metabolites.
			rs433598	The homozygous CC genotype—a biomarker for a high risk of impaired VPA acetylation and increased levels of toxic metabolites.

Note: CYP—Cytochrome; UGT—Uridine diphosphate glucosyltransferase; ACADSB—Acyl-Coenzyme A dehydrogenase short/branched chain; ACSM1- Acyl-Coenzyme A synthetase medium chain family member 1; CoA—Coenzyme A; UDP- Glucuronosyltransferase.
